# Supplementary figures and images for: New advances in jellyfish anatomy: the benefits of endocasts and X-ray microtomography in the investigation of the gastrovascular system of Cotylorhiza tuberculata (Scyphozoa; Rhizostomeae; Cepheidae)
Source: PLoS One. 2025 Nov 14;20(11):e0336682. doi: 10.1371/journal.pone.0336682 (PMC12617847; doi:10.1371/journal.pone.0336682)

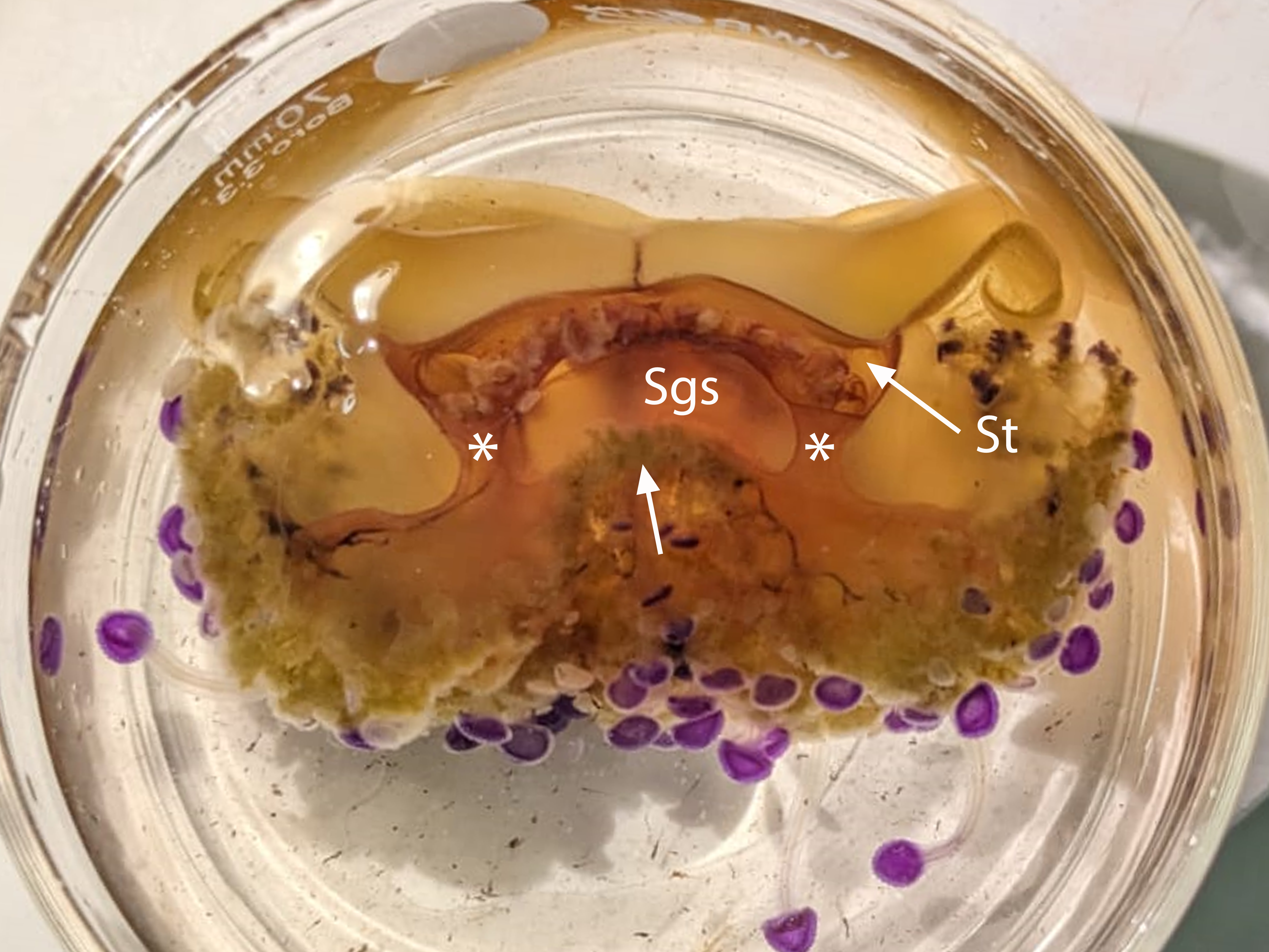

Supplement: S1 Fig — Visible the Subgenital sinus (Sgs), two of the subgenital ostia (Asterisks), the stomach (St) with part of the gonads. Arrow indicates the area containing the complex of central branchings emerging from the 2-4-8 central canal system (schematized in S2 Fig.), including the future brood-carrying filaments and some smaller club-shaped digitations. (TIF) [file pone.0336682.s001.tif]

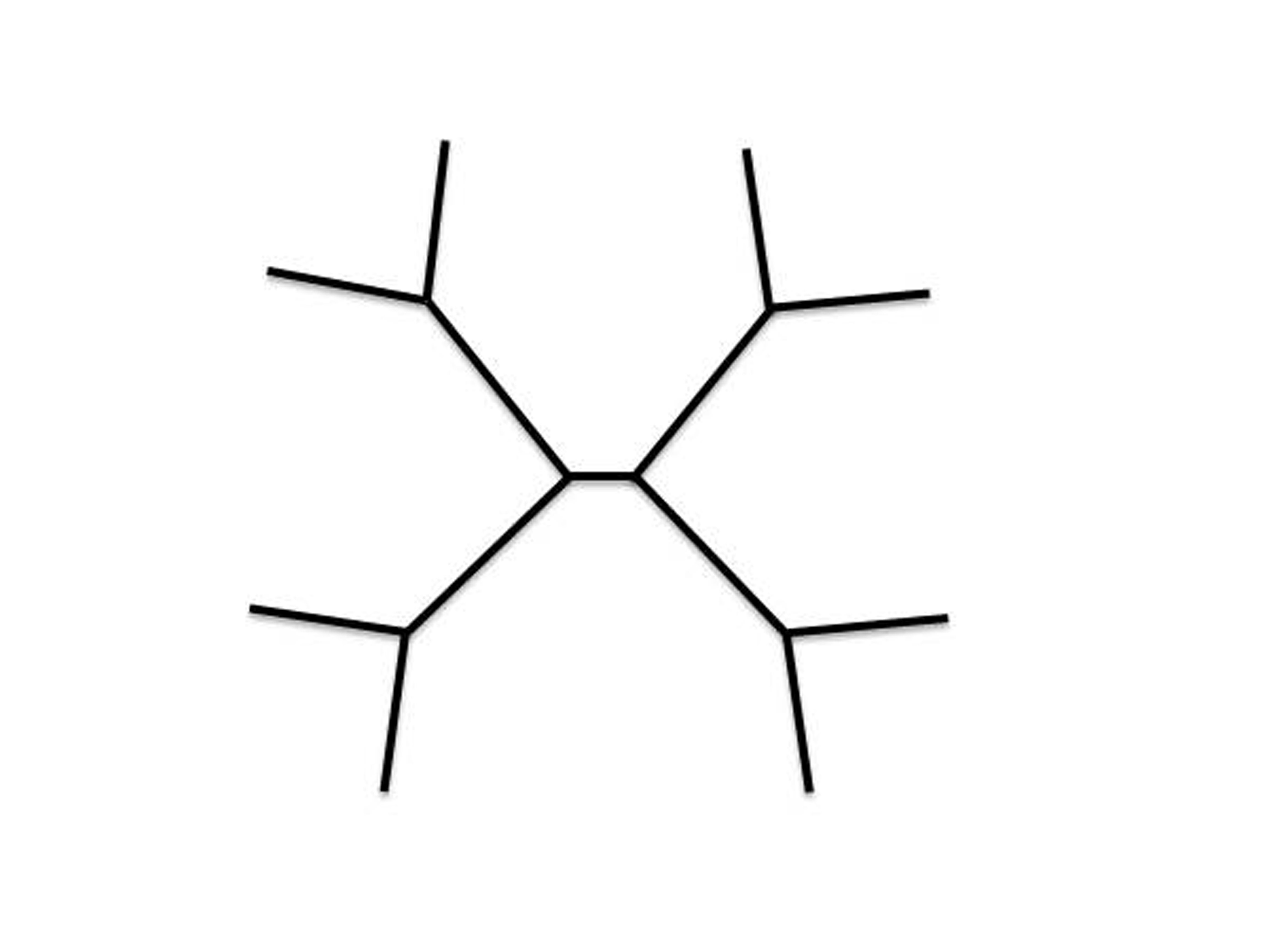

Supplement: S2 Fig — (TIF) [file pone.0336682.s002.tif]

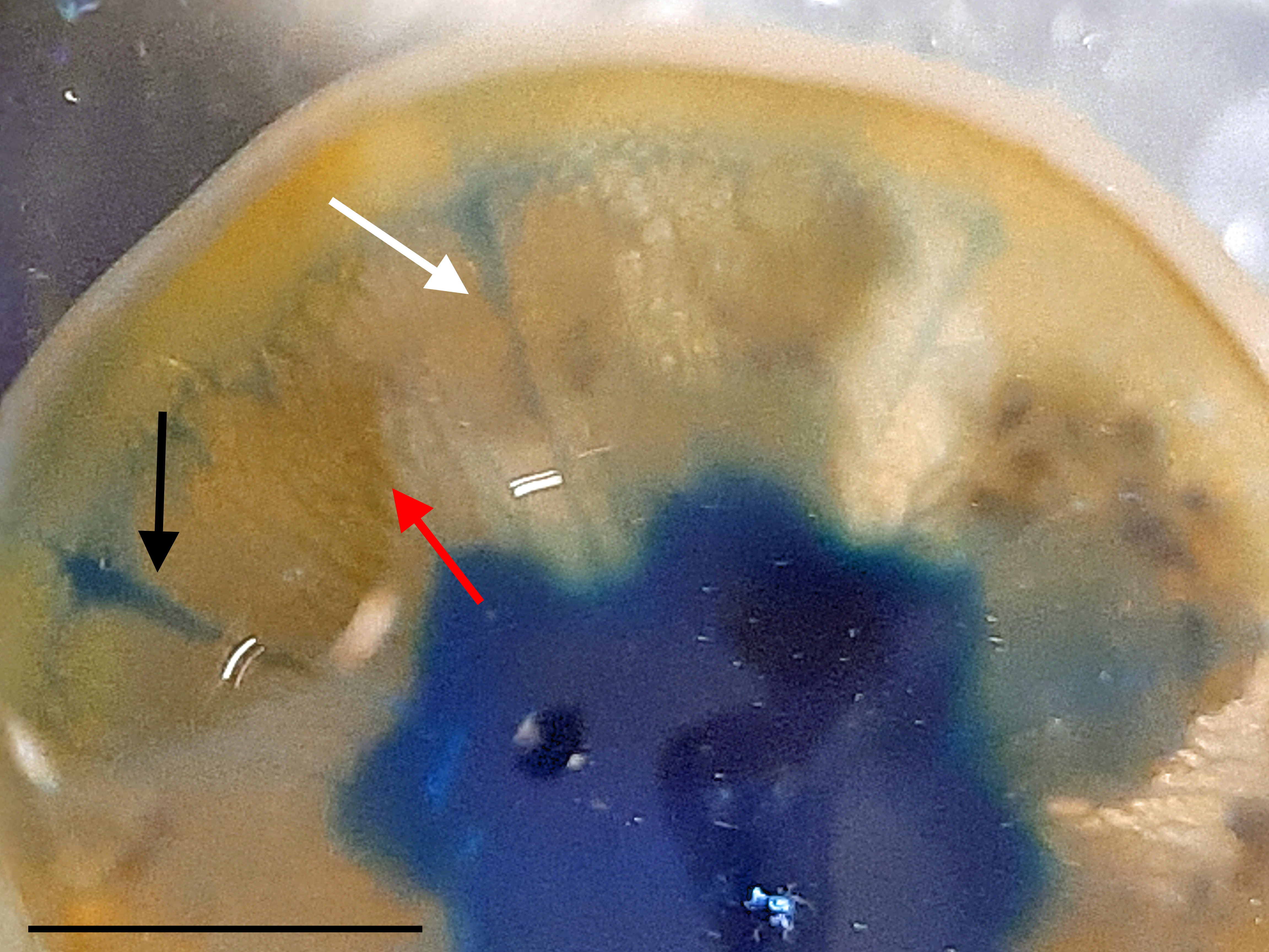

Supplement: S3 Fig — Exumbrellar view. Evident the stain present into the stomach, the per- and interradial canals (white and black arrows, respectively) and into the “pseudo ring canal complex” (See text). The web of adradial canals is now faded (red arrow). The outermost, distal part of the anastomoses was practically unstained, apart from a small area adjacent to the perradial canal on the left. (IFF) [file pone.0336682.s003.tiff]

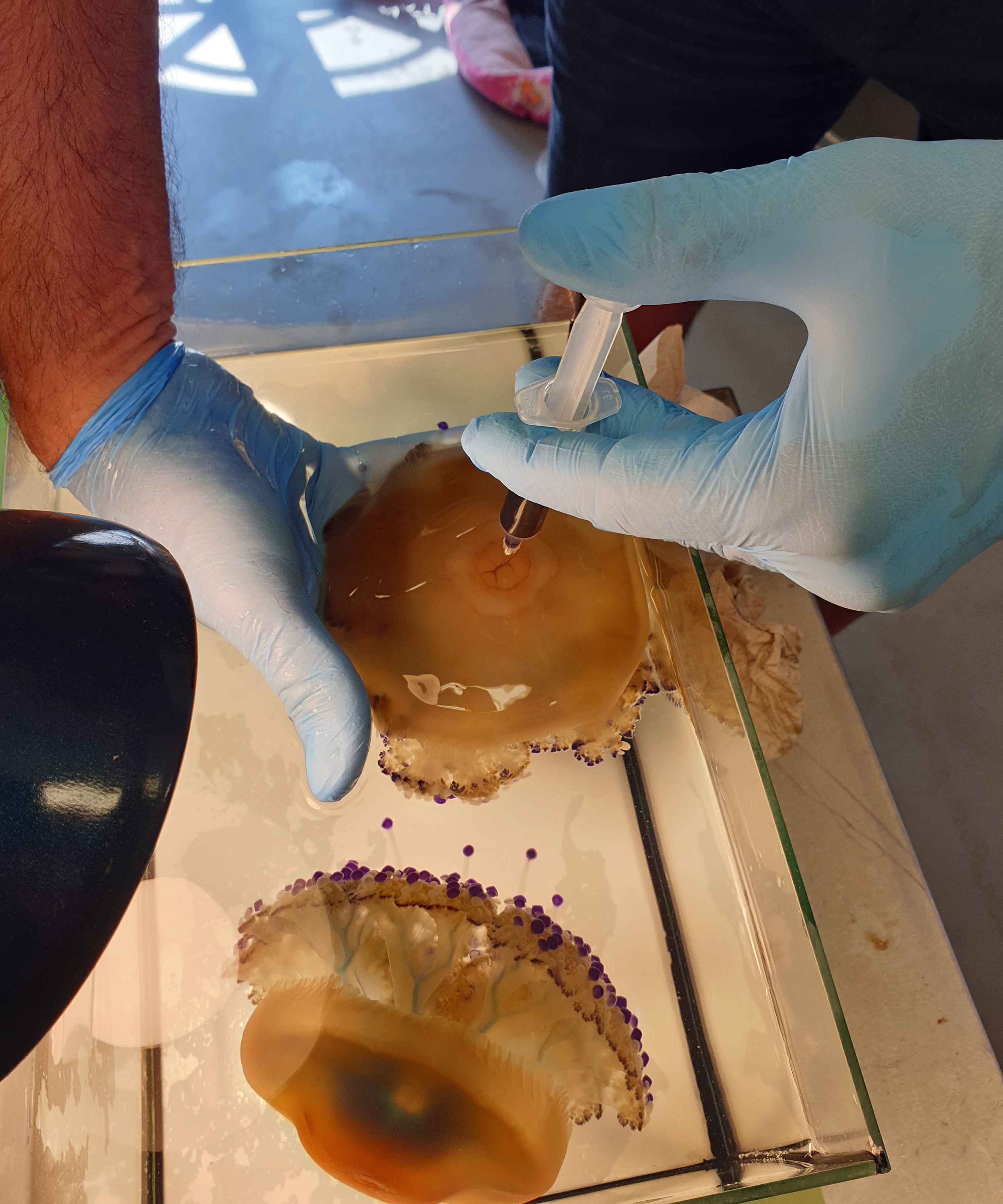

Supplement: S4 Fig — Two living specimens, one at the moment of the injection into the stomach, the second after the injection. (TIF) [file pone.0336682.s004.tif]
