# Supplementary material for: New advances in jellyfish anatomy: the benefits of endocasts and X-ray microtomography in the investigation of the gastrovascular system of Cotylorhiza tuberculata (Scyphozoa; Rhizostomeae; Cepheidae)
Source: PLoS One. 2025 Nov 14;20(11):e0336682. doi: 10.1371/journal.pone.0336682 (PMC12617847; doi:10.1371/journal.pone.0336682)
Supplement: Attachment — Submitted filename: PONE-D-25-22492_Review.pdf [file pone.0336682.s010.pdf]

PLOS ONE

New advances in jellyfish anatomy: the benefits of endocasts and X-ray microtomography in the investigation of the gastrovascular system of Cotylorhiza tuberculata (Scyphozoa; Rhizostomeae; Cepheidae).

--Manuscript Draft--

**Manuscript Number:**	PONE-D-25-22492
**Article Type:**	Research Article
**Full Title:**	New advances in jellyfish anatomy: the benefits of endocasts and X-ray microtomography in the investigation of the gastrovascular system of Cotylorhiza tuberculata (Scyphozoa; Rhizostomeae; Cepheidae).
**Short Title:**	Anatomy of Cotylorhiza tuberculata via endocast and X-ray µCT
**Corresponding Author:**	Gregorio Motta<br>University of Trieste: Universita degli Studi di Trieste<br>trieste, ITALY
**Keywords:**	Cotylorhiza tuberculata; jellyfish; jellyfish anatomy; jellyfish physiology; gastrovascular system; digestive system evolution; X-ray microtomography; resin endocast
**Abstract:**	Historically, research on jellyfish has been viewed as secondary in importance and has not benefited from technical advances that could improve the quality of the results when compared to other disciplines. The most notable example is the anatomical research on jellyfish, which has been done using conventional methods for many years. Thus far, recent studies have shown that X-ray microtomography (µCT) and resin endocasts can yield outputs with remarkably high detail quality. The application of a similar protocol to Cotylorhiza tuberculata has allowed us to redescribe the anatomy of this species' gastrovascular system, adding numerous details, such as the double constricted canal structure present in the oral arms, which was absent in previous descriptions. Additionally, functional anatomy experiments have revealed a double circulation system within these canals, featuring specialized oral arms' openings for intake and outflow, as previously observed in Rhizostoma pulmo. These findings challenge the theory of a simple digestive system in cnidarians. Given the genetic distance between Cotylorhiza tuberculata and Rhizostoma pulmo, we propose that this complex digestive system may be more widespread among the Rhizostomeae, suggesting that jellyfish are more complex organisms than previously thought.
**Order of Authors:**	Gregorio Motta
	Marco Voltolini
	Lucia Mancini
	Diego Dreossi
	Francesco Brun
	Valentina Tirelli
	Lorenzo Peter Castelletto
	Manja Rogelja
	Antonio Terlizzi
	Massimo Avian
**Additional Information:**	
**Question**	**Response**
**Financial Disclosure**<br><br>Enter a financial disclosure statement that	Yes

describes the sources of funding for the work included in this submission. Review the submission guidelines for detailed requirements. View published research articles from *PLOS ONE* for specific examples.<br><br>This statement is required for submission and **will appear in the published article** if the submission is accepted. Please make sure it is accurate.<br><br>**Funded studies**<br>Enter a statement with the following details:<br>• Initials of the authors who received each award<br>• Grant numbers awarded to each author<br>• The full name of each funder<br>• URL of each funder website<br>• Did the sponsors or funders play any role in the study design, data collection and analysis, decision to publish, or preparation of the manuscript?<br><br>Did you receive funding for this work?	
Please add funding details.<br>as follow-up to "**Financial Disclosure**<br><br>Enter a financial disclosure statement that describes the sources of funding for the work included in this submission. Review the submission guidelines for detailed requirements. View published research articles from *PLOS ONE* for specific examples.<br><br>This statement is required for submission and **will appear in the published article** if the submission is accepted. Please make sure it is accurate.	This work was partially funded by the National Biodiversity Future Centre (NBFC) Program, Italian Ministry of University and Research, PNRR, Missione 4 Componente 2 Investimento 1.4 (Project: CN00000033) to A.T. The funder had no role in study design, data collection and analysis, decision to publish, or preparation of the manuscript

Powered by Editorial Manager® and ProduXion Manager® from Aries Systems Corporation

**Funded studies**<br>Enter a statement with the following details:<br>• Initials of the authors who received each award<br>• Grant numbers awarded to each author<br>• The full name of each funder<br>• URL of each funder website<br>• Did the sponsors or funders play any role in the study design, data collection and analysis, decision to publish, or preparation of the manuscript?<br><br>Did you receive funding for this work?"	
Please select the country of your main research funder (please select carefully as in some cases this is used in fee calculation).<br>as follow-up to "**Financial Disclosure**<br><br>Enter a financial disclosure statement that describes the sources of funding for the work included in this submission. Review the submission guidelines for detailed requirements. View published research articles from *PLOS ONE* for specific examples.<br><br>This statement is required for submission and **will appear in the published article** if the submission is accepted. Please make sure it is accurate.<br><br>**Funded studies**<br>Enter a statement with the following details:<br>• Initials of the authors who received each award<br>• Grant numbers awarded to each author<br>• The full name of each funder<br>• URL of each funder website<br>• Did the sponsors or funders play any role in the study design, data collection and analysis, decision to publish, or preparation of the manuscript?<br><br>Did you receive funding for this work?"	ITALY - IT
**Competing Interests**	The authors have declared that no competing interests exist.

Use the instructions below to enter a competing interest statement for this submission. On behalf of all authors, disclose any competing interests that could be perceived to bias this work—acknowledging all financial support and any other relevant financial or non-financial competing interests.<br><br>This statement is required for submission and **will appear in the published article** if the submission is accepted. Please make sure it is accurate and that any funding sources listed in your Funding Information later in the submission form are also declared in your Financial Disclosure statement.<br><br>View published research articles from *PLOS ONE* for specific examples.<br><br>**NO authors have competing interests**<br><br>Enter: *The authors have declared that no competing interests exist.*<br><br>**Authors with competing interests**<br><br>Enter competing interest details beginning with this statement:<br><br>*I have read the journal's policy and the authors of this manuscript have the following competing interests: [insert competing interests here]*<br><br>* typeset	
**Ethics Statement**<br><br>Enter an ethics statement for this submission. This statement is required if the study involved:<br><br>• Human participants<br>• Human specimens or tissue<br>• Vertebrate animals or cephalopods<br>• Vertebrate embryos or tissues	All research present in the manuscript (being on jellyfish) did not require ethical approval.

Powered by Editorial Manager® and ProduXion Manager® from Aries Systems Corporation

• Field research

Write "N/A" if the submission does not require an ethics statement.

General guidance is provided below. Consult the submission guidelines for detailed instructions. **Make sure that all information entered here is included in the Methods section of the manuscript.**

**Format for specific study types**<br><br>**Human Subject Research (involving human participants and/or tissue)**<br>• Give the name of the institutional review board or ethics committee that approved the study<br>• Include the approval number and/or a statement indicating approval of this research<br>• Indicate the form of consent obtained (written/oral) or the reason that consent was not obtained (e.g. the data were analyzed anonymously)<br><br>**Animal Research (involving vertebrate animals, embryos or tissues)**<br>• Provide the name of the Institutional Animal Care and Use Committee (IACUC) or other relevant ethics board that reviewed the study protocol, and indicate whether they approved this research or granted a formal waiver of ethical approval<br>• Include an approval number if one was obtained<br>• If the study involved *non-human primates*, add *additional details* about animal welfare and steps taken to ameliorate suffering<br>• If anesthesia, euthanasia, or any kind of animal sacrifice is part of the study, include briefly which substances and/or methods were applied<br><br>**Field Research**<br><br>Include the following details if this study involves the collection of plant, animal, or other materials from a natural setting:<br>• Field permit number<br>• Name of the institution or relevant body that granted permission	
**Data Availability**<br><br>Authors are required to make all data underlying the findings described fully available, without restriction, and from the time of publication. PLOS allows rare exceptions to address legal and ethical concerns. See the PLOS Data Policy and FAQ for detailed information.	Yes - all data are fully available without restriction

Powered by Editorial Manager® and ProduXion Manager® from Aries Systems Corporation

A Data Availability Statement describing where the data can be found is required at submission. Your answers to this question constitute the Data Availability Statement and **will be published in the article**, if accepted.<br><br>**Important:** Stating 'data available on request from the author' is not sufficient. If your data are only available upon request, select 'No' for the first question and explain your exceptional situation in the text box.<br><br>Do the authors confirm that all data underlying the findings described in their manuscript are fully available without restriction?	
**Describe where the data may be found in full sentences. If you are copying our sample text, replace any instances of XXX with the appropriate details.**<br><br>• If the data are **held or will be held in a public repository**, include URLs, accession numbers or DOIs. If this information will only be available after acceptance, indicate this by ticking the box below. For example: *All XXX files are available from the XXX database (accession number(s) XXX, XXX.*).<br>• If the data are all contained **within the manuscript and/or Supporting Information files**, enter the following: *All relevant data are within the manuscript and its Supporting Information files.*<br>• If neither of these applies but you are able to provide **details of access elsewhere**, with or without limitations, please do so. For example:<br><br>*Data cannot be shared publicly because of [XXX]. Data are available from the XXX Institutional Data Access / Ethics Committee (contact via XXX) for researchers who meet the criteria for access to confidential data.*<br><br>*The data underlying the results presented in the study are available from (include the name of the third party*	All relevant data are within the manuscript and its Supporting Information files

and contact information or URL).<br>• This text is appropriate if the data are owned by a third party and authors do not have permission to share the data.<br><br>* typeset	
Additional data availability information:	

Powered by Editorial Manager® and ProduXion Manager® from Aries Systems Corporation

Manuscript

Click here to access/download;Manuscript;Motta_Cotylorhiza_Plos.docx

New advances in jellyfish anatomy: the benefits of endocasts and X-ray microtomography in the investigation of the gastrovascular system of *Cotylorhiza tuberculata* (Scyphozoa; Rhizostomeae; Cepheidae).

Anatomy of *Cotylorhiza tuberculata* via endocast and X-ray µCT

Gregorio Motta[1,2*], Marco Voltolini[3], Lucia Mancini[4], Diego Dreossi[5], Francesco Brun[6], Valentina Tirelli[7,8], Lorenzo Peter Castelletto[1], Manja Rogelja[9], Antonio Terlizzi[1,2,8], Massimo Avian[1].

[1] Department of Life Science, University of Trieste, Trieste, Italy

[2] Department of Integrative Marine Ecology, Stazione Zoologica Anton Dohrn, Napoli, Italy

[3] Department of Earth Science Ardito Desio, University of Milano, Milano, Italy

[4] Slovenian National Building and Civil Engineering Institute, Ljubljana, Slovenia

[5] Elettra-Sincrotrone Trieste S.C.p.A., Basovizza, Trieste, Italy

[6] Department of Engineering and Architecture, University of Trieste, Trieste, Italy

[7] National Institute of Oceanography and Applied Geophysics, Trieste, Italy

[8] National Biodiversity Future Center (NBFC), National Biodiversity Future Center, Palermo, Italy

[9] University of Primorska, Aquarium Piran, Piran, Slovenia

* Corresponding author

E-mail: gregorio.motta@phd.units.it (GM)

Abstract

Historically, research on jellyfish has been viewed as secondary in importance and has not benefited from technical advances that could improve the quality of the results when compared to other disciplines. The most notable example is the anatomical research on jellyfish, which has been done using conventional methods for many years. Thus far, recent studies have shown that X-ray microtomography (µCT) and resin endocasts can yield outputs with remarkably high detail quality. The application of a similar protocol to *Cotylorhiza tuberculata* has allowed us to redescribe the anatomy of this species' gastrovascular system, adding numerous details, such as the double constricted canal structure present in the oral arms, which was absent in previous descriptions.

Additionally, functional anatomy experiments have revealed a double circulation system within these canals, featuring specialized oral arms' openings for intake and outflow, as previously observed in *Rhizostoma pulmo*. These findings challenge the theory of a simple digestive system in cnidarians. Given the genetic distance between *Cotylorhiza tuberculata* and *Rhizostoma pulmo*, we propose that this complex digestive system may be more widespread among the Rhizostomeae, suggesting that jellyfish are more complex organisms than previously thought.

Introduction

Medusozoa (phylum Cnidaria) is one of the oldest group of metazoans, whose fossils date back over 500 million years [1]. Despite their long evolutionary history, they are still relatively understudied compared to other marine organisms [2,3]. The first scientific studies on jellyfish can be traced back to the 18th and 19th centuries and focused on taxonomy and basic morphology [4–6]. In recent decades, a new wave of studies on their physiology, behavior, and ecological roles has emerged due to their increasing abundance and impact on ecosystems and human activities [2,3,7–11]. Despite this recent increase in scientific interest, general knowledge of jellyfish remains limited compared to other taxonomic groups, and many aspects of their biology and ecology are still poorly understood [12,13]. Numerous jellyfish species have received limited scientific attention and the description of their anatomy and physiology are largely undocumented; moreover, much of the existing knowledge is based on a few well-known species often based on old-fashioned research. This state of jellyfish research collides with other scientific fields where studies and references are continually being updated [12].

Jellyfish zoology may be one of the few research areas where updates to techniques and instruments have not been adequately considered, missing the opportunity to make significant advances [14]. Such updates could help address these persistent limitations and provide new and more detailed descriptions that can support or challenge the validity of older reports.

Most of research now focus on the "external" functioning of jellyfish as how their presence impact ecosystems or how oceanographic conditions affect jellyfish abundance and distributions [8–10,15–17]. Despite this knowledge is expanding, a proper "internal" description of anatomy and physiology is missing for most of the species, and this is one of the most important deficiencies to address. New technologies could help circumvent historical problems in anatomical and zoological studies, as the opacity and thickness of the mesoglea layer severely compromising the quality and accuracy of traditional observations. Advances in anatomical studies such that of Avian and

colleagues [18], who proposed a brand-new use of endocasts and microtomography for the three-dimensional analysis of the gastrovascular system of jellyfish, have significantly overtaken traditional analyses and the classic bibliography made of two-dimensional representations that lack many details. In addition, Presnell [19] and Avian and colleagues [18] reported astonishing results in functional anatomy that challenged old paradigms such as the gut-sac digestive cavity in Cnidaria and Ctenophora and provided evidence for more complex digestive systems, albeit only appearing in much more evolved organisms [20–22]. The prevailing theories on the evolution of metazoan assume that Cnidaria and Ctenophora have simple, single-cavity digestive systems [23], while the first through-gut appeared in Bilateria [24,25]. Thus, new discoveries in jellyfish research may not be limited to Cnidaria, but may also support or discuss broader issues such as the overarchingly accepted evolutionary theory of the development digestive system in Animalia.

The order Rhizostomeae certainly includes a group of taxa that are among the most representative in the coastal macro-megaloplankton of all seas, both in terms of size (up to a few meters) and occasional abundance (the so-called jellyfish blooms) [26,27]. *Cotylorhiza tuberculata* (Rhizostomeae, Kolpophorae, Cepheidae), usually blooming during summer [28], is a common species in the Mediterranean Sea [29–33] which hosts photosynthetic symbionts that play a fundamental role fin its survival [32,34]. These jellyfish may have a potential top-down effects on planktonic communities by selectively feeding on diatoms, ciliates, larvae of some mollusks and copepods [33]. In this work, we chose *C. tuberculata* as a target species, as it is widely distributed for both its abundance and distribution in the Mediterranean Sea and its genetic distance [35] with *Rhizostoma pulmo* (extensively redescribed by Avian and colleagues [18]), to investigate possible similarities and differences in anatomy and function. Our aim was to (1) test whether the protocol developed and proposed by Avian and colleagues [18] for *R. pulmo* (resin endocasts followed by X-ray computed microtomography (μCT)) is applicable to other species to test its flexibility and versatility; (2) describe the anatomy and functional anatomy of this species in three dimensions and great detail; (3) test whether the recently discovered double canal circulation [18] and opening

differentiations are unique to *R. pulmo* or they are common to other species (demonstrating a strong bias due to old techniques that could not provide detailed anatomical data).

Results

Morphological analyses

Using dissections, contrast injections, resin casts, and X-ray μCT, we were able to produce an updated and very detailed three-dimensional description of the gastrovascular structure of *Cotylorhiza tuberculata* (Fig 1).

Fig 1. *Cotylorhiza tuberculata*, adult specimen approximately 30 cm in diameter. Photo taken in the WWF Miramare Marine Protected Area, Trieste, Italy (Miramare Castle in the background). Photo courtesy of Adriano Morettin.

Umbrella and stomach

The central cavity of *C. tuberculata* is usually described as circular, but its shape is indeed more complex. The upper part of the stomach, just below the thicker central part of the exumbrella, is effectively circular, and symmetric, with a convexity facing the exumbrella. The lower part, however, is provided with four distal, perradial, kidney-shaped protuberances that have lateral concavities (Fig 2A-2C). These protrusions partially extend in the arm disk, into the subgenital sinus space (Fig 2B), each one containing the more lateral parts of the four interradial folded gonads with proximal gastric cirri stripes. The floor of the stomach consists of a very thin membrane and there is no central opening. In the center of the stomach floor, the surface has a Y-shaped relief structure, with opposite bifurcations clearly visible. Further V-shaped folds are visible at its distal apex, (Fig 2B, black arrow). The canals that communicate with the manubrium are eight, flattened and originate under the lateral extensions of the gonads (Fig 2B-2D).

Below the stomach is the subgenital sinus (or cavity), a single cruciform cavity, with four ovoid to heart-shaped interradial ostia (in this case the apex is exumbrellar) (Fig 2E, 2F; S1 Fig, asterisks), alternating with four perradial basal pillars which contribute to the octagonal shape of the oral disk.

Fig 2. Anatomy of the stomach and subgenital sinus. (**A**) Dissection of a stomach at the level of the four kidney-shaped protrusions, subumbrellar view. Arrow indicates one of the interradial canals. Scale bar = 6 cm. (**B**) Endocast of one stomach, subumbrellar view. Red arrows indicate two of the oral arms canals. Black arrow indicates the scar present in the central area. Scale bar = 2.25 cm. (**C**) Two young living specimens with stained stomachs (Methylene blue and Neutral red stained, respectively). Scale bar = 10 cm. (**D**) Siliconic endocast of the subgenital sinus of the same specimen of (**C**). Exumbrellar view. Arrows indicate two of the exit points of the oral canals. Asterisks indicate the thin extensions corresponding to the stomach's protrusions in (**C**). The octagonal edge is an artifact due to the shape of the container used. Scale bar = 2.5 cm. (**E**) Adradial canals stained with toluidine blue, subumbrellar view. Interradius (and one of the subgenital ostia, asterisk) just in the middle of the picture. Scale bar = 0.5 cm. (**F**) Specimen stained with toluidine blue, arrow indicates the subgenital ostium. Scale bar = 4 cm.

Many attempts have been made to reproduce the three-dimensional shape of both the subgenital sinus and the stomach and their volumetric relationships. We tried to inject resin with a syringe both into the stomach and through the ostia into the subgenital sinus. Many attempts were unreliable because when the resin was first injected into the stomach, it swelled and compressed the subgenital sinus (the membrane separating the stomach from the sinus is extremely thin and plastic). Similarly, when the resin was injected into the subgenital sinus, we obtained an inflated subgenital sinus, thick, circular and not cruciform and the stomach was squeezed.

The most effective way to reconstruct and to maintain the correct volumetric ratio of the stomach-subgenital sinus (Fig 2C, 2D) was to remove the umbrella above the stomach and carefully pour the resin (to avoid the pressure of the syringe) and then, once polymerization had taken place, pour a silicone resin through the ostia into the sinus. It is interesting to note that the cast of the subgenital sinus shows a greater cruciform thickness, but there are four flat and thin expansions between the cruciform arms (Fig 2D, asterisks), with eight clefts corresponding to the origins of the oral arm canals (Fig 2D, arrows). This suggests that the subgenital cavity is reminiscent of the theoretical

circular shape, with its four cruciform pillars alternating with the four kidney-shaped protrusions of the stomach resting on the floor of the subgenital sinus.

Umbrellar canal system

The umbrellar canal system consists of 4 perradial and 4 interradial (all rhopaliar) canals and a number of adradial canals varying between 7 and 10-11 per octant, depending on the size of the specimen (Fig 3A).

Fig 3. Dissection of a stomach immediately below the origin of the umbrellar canals. (**A**) Subumbrellar view. The red arrow indicates one of the perradial canals, the blue one the interradial canals, the black ones the adradial canals. Scale bar = 1 cm. (**B**) Detail of (**A**), showing a pair of adradial canals (arrows) with a single origin (asterisk). Scale bar = 1 mm. (**C**) An octant with the adradial canals stained with toluidine blue (white asterisk = perradial canal, black asterisk =interradial canal). Scale bar = 2.5 cm. The square brackets show the widening of some canals and the corresponding canal network, which simulates a kind of "pseudo ring canal".

Counting the adradial canals, which originate from the stomach at the point of contact between the upper circular portion and the lower portion with the four kidney-shaped extensions, is very difficult due to the frequent dichotomies (Fig 3B) and/or ramifications. Therefore, we decided to count only the openings of the canals on the sides of the stomach (Fig 3A, black arrows). All canals are strongly flattened at the ex-subumbrellar plane, and highly anastomosed along their entire length up to the edge of the umbrella, with no evident ring canal. The numerous anastomoses begin a few millimeters from their origin, with the exception of the interradial canals, which have no connections in the proximal part (less than 1 cm in the largest specimens). Both the perradial and interradial canals show an expansion in width after about 1 cm from the origin, which then decreases in the last distal third (Fig 3, blue and red arrows, respectively). According to the current state of the art, the umbrellar canals pattern of *C. tuberculata* is described as having no annular (or ring) canals. We have not detected the presence of a distal annular canal. However, approximately in the distal half to two-thirds of the canal system, we observed a circular, irregular, and sometimes larger, perpendicular canal (connected to the anastomosis network) running along the entire

umbrella, thus resembling an inner pseudo ring canal (Fig 3C). Some zooxanthellae clusters are detectable in correspondence with the lateral margins, or blind-ended branches, of the canals.

Manubrium canal system

Eight canals originate from the floor of the stomach next to the most lateral portions of the gonads (Fig 2B; 4A, 4B; 5A, 5B). In section they are symmetrical, flattened, with a maximum diameter of a few millimeters, with two slight terminal dilatations (Fig 5A, 5B; 6A-6C).

Fig 4. Origin of the oral arm canal from the stomach. Magnification of the stomach floor just under the lateral end of a gonad, slightly contrasted with toluidine blue, exumbrellar view. (**A**) A full opening, the portion of the gonad has been previously displaced. The arrows point to the slightly wider ends. (**B**) The same of (**A**), with a needle partially inserted into the opening. Scale bars = 2 mm.

Fig 5. *Cotylorhiza tuberculata* gastrovascular system of the manubrium. (**A**) Resin endocast of the whole gastrovascular system of a specimen of 35 cm in diameter. (**B**) 3D rendering obtained by X-ray microtomographic data of the resin endocast showed in (**A**). Tomographic reconstruction. the arrows in both (**A**) and (**B**) indicate the point of bifurcation of the two distal second-order branches.

Immediately after emerging downward from the subgenital sinus, they expand considerably and even reach a diameter of several cm in larger specimens (Fig 4A, 4B; 5A, 5B). In section, one canal is tubular distally, and very flattened in the proximal inner/lower part (Fig 6A-6C).

Fig 6. Sections of the oral arms. Sections performed in the proximal portions, before the first bifurcations. Specimen with a diameter of 17 cm. (**A**) Section through an oral arm canal, lateral portion on the left. The black arrows indicate the total extension of one canal, the red arrows indicate an area of adhesion. Scale bar = 0.5 cm. (**B**), the same of (**A**), from a specimen of 20 cm in diameter, slightly contrasted with toluidine staining. Lateral part top right. Arrows as in (**A**). Scale bar = 1 cm. (**C**) Another section from the specimen of (**B**), lateral portion on the left. White arrows as in (**B**), the red arrows indicate the wrinkled area. Scale bar = 1 cm.

When analyzing the transverse sections performed in the proximal part of the oral arms, it was possible to observe a roughly median area where the two stripes of gastrodermis are in contact (Fig 6A, 6B, red arrows), but this adhesion does not seem to be structurally stable, as sections with a patent lumen were also observed in this area and the two gastrodermic sides can be easily separated with a needle. However, the gastrodermis median area (the adhering stripe) has a wrinkled

appearance, which could indicate adaptation in this area for increased adhesiveness (Fig 6A-6C, red arrows). The simulation of flow velocity performed on one of the oral arms virtually isolated from the skeletonization (Fig 7C), highlighted an area of high flow speed on the outer side of the oral arm canal, followed by the inner side, while the median stripe is clearly visible (there is a single point, probably where the two layers are not in contact, only where the flow velocity is equal to that of the lateral sides, Fig 7C, arrow).

Fig 7. Endocasts and skeletonized oral arm. (**A**) Detail of the endocast of Fig 5. The arrows indicate the branches of Ist, IInd and IIIrd order (white, red, and yellow respectively). (**B**) Another oral arm endocast of a 30 cm diameter specimen. The asterisks indicate the presence of anastomoses between the lateral median branches. (**C**) A skeletonized oral arm of the specimen in (**A**), where the flow velocity (red = faster, blue = lower) was simulated. The arrow indicates a point of a relatively higher flow point between the two lateral portions.

The eight oral arms and their associated canals run intact for about half their length in the larger jellyfish, and about 70-75% in the smaller jellyfish (15 cm in diameter), after which they are divided into a total of 16 distal portions of the oral arms. Each distal portion can lead to a second and a third order dichotomization (Fig 7A). Ramifications that extend along the entire oral arms, originate from the flattened part of the canals, may be partially anastomosed (Fig 7B) and all develop on the inner/lower side of the manubrium. All these branches may terminate in many openings or in club-shaped and sucker-shaped ends containing blind-ended canals with a white or purple/violet colored tubercle (the distal part) (Fig 1). Some of these club- or sucker-shaped ends, usually purple, can be several centimeters long.

This resin endocast also revealed the finest features of the manubrium and the oral arm canals (Fig 5A, 5B; 7A, 7B). In the central area of the manubrium, just below the subgenital sinus, there is a peculiar canal system connecting all eight canals of the oral arms. From a subumbrellar view, a structure can be recognized that consists of two "specular Y-shaped" canals that are connected at the base (see S2 Fig). The four lateral branches then dichotomize further to form eight canals that merge with the proximal parts of the oral arm canals. All these canals are drop-shaped in section in

younger specimens (Fig 8A-8C), in older individuals they mirror the shape of the canals of the oral arms on a small scale, with a thicker exumbrellar part and a flattened subumbrellar part (Fig 8E). This structure with one to four to eight central canals was observed in all jellyfish analyzed, regardless of size and sex.

Fig 8. Central area of the manubrium. (**A**) Endocast of a specimen with a diameter of 13 cm, showing the central canal system with 1-4-8 canals, subumbrellar view. The arrows indicate two of the eight lateral branches. (**B**) 3D rendering of the endocast from (**A**). The virtual cutting plane runs exactly through the central canal system. Exumbrellar view. (**C**) The same 3D rendering of (**B**), but in subumbrellar view. The inner part is still free of branches. (**D**) Section of the central area of the manubrium of a specimen with a diameter of 28 cm, slightly contrasted with toluidine blue, in subumbrellar view. Several branches and club-shaped digitations are recognizable. The arrows indicate two of the eight canals. (**E**) The same 35 cm diam. area of the endocast, in lateral view. The arrow indicates the median canal, carrying branches (upside-down view). (**F**) 3D rendering of the endocast in (**E**), exumbrellar view. The arrows indicate two of the eight distal ramifications.

These double-Y canals have a few protrusions in adults, many of which end blindly and insert into club-shaped digitations, whereas no protrusions were found in the endocast of the younger and smaller specimen (Fig 8A-8C). These digitations vary greatly in length, from a couple of millimeters to a centimeter or more (Fig 8D-8F).

Image processing and analysis of the X-ray µCT data

The most complete endocasts of three specimens with a diameter of 13, 15 and 34 cm, respectively, were visualized using virtual sectioning and 3D rendering procedures. The X-ray µCT data allowed the investigation of the less accessible areas of the gastrovascular system that are difficult to be properly examined by visual observations, such as the pattern of the central canal system (Fig 8). This approach confirmed the regularity of this structure in all specimens we analyzed.

Morphometric analysis of microtomographic data

Topological characterization by skeletonization and skeleton analysis has been carried out on the best reproduced single oral arm for two specimens (15 and 34 cm in diameter), as the smaller one (13 cm in diameter) had no oral arm completely filled with resin (Fig 9, S1, S2, S3 Video).

Table 1 summarized the most important data of the skeleton analysis. There we have considered as endpoints are all the terminations of the canals. It should be noted that in the adult specimen multiple openings were "connected" by artifact single droplets of resin, which were then counted as single endpoints by the software. However, in most cases, it was observed that the droplet connected only two endpoints, therefore the number of openings is slightly underestimated.

Fig 9. Quantitative analysis on the gastrovascular system. (**A**) 3D rendering of the entire cast of the gastrovascular system of the young specimen (diameter 15 cm) measured via X-ray µCT. (**B**) Isosurface rendering showing the superimposed (red) segmented gastrovascular system of the entire manubrium. (**C**) 3D rendering of the entire cast of the gastrovascular system of the fully developed specimen (diameter 35 cm) measured by X-ray µCT. (**D**) Isosurface rendering showing the overlied (red) segmented gastrovascular system of the entire manubrium (pale blue, exumbrellar view). (**E**) Volume rendering of a single oral arm (yellow) with overlaid isosurface rendering of the segmented gastrovascular system (red) in the young specimen. (**F**) As in (**E**), in the fully developed specimen.

Table 1. Summary of the skeleton analysis of a single oral arm of a young (diameter 15 cm) and fully developed (diameter 35 cm) specimen.

	**15 cm diam.**	**35 cm diam.**
Average branch length [mm]	2.59	4.32
Average branch Euclidean Distance [mm]	2.06	3.51
ED/Length	0.80	0.81
Number of total branches	411	1442
Number of total junctions	224	688
Number of total endpoints	119	704

ED = Euclidean distance.

Comparing the data between the young and adult oral arm (Fig 9E, 9F), there is a clear increase in all the data obtained, with the exception of the ED/length ratio, which remains virtually unchanged. This stable ED/length ratio in *C. tuberculata* suggests that branching geometry is determined early in development and scales proportionally with size.

As far as the number of endpoints is concerned, we estimated that a jellyfish (with 8 oral arms) has about 952 endpoints when it is young (specimen with a diameter of 15 cm) and 5632 when it is fully developed (specimen with a diameter of 35 cm), taking into account that the data were obtained by multiplying the endpoints found in a single oral arm (Tab. 1). These calculations may overestimate the number of actual endpoints as not all endpoints are to be considered as openings to the outside (the so-called generic "mouthlets"), as the computation also included the blind ends of the club-shaped appendages (at least one hundred per oral arm in adult jellyfish) and, in the central part, of the brood carrying filaments.

Functional Anatomy

Experiments in which small amounts of stained solutions were injected into the stomach (S3 Fig) of young living specimens (diameter between 10 and 15 cm) have consistently shown that the outflow affects only the most lateral, slightly dilated part of the oral arm canals (Fig 10A-10C; S4 and S5 Video). The inner elongated, flattened parts are not involved in the outflow (apart from some artifacts due to incorrect injection of the dye into the stomach).

Fig 10. Functional anatomy of the gastrovascular system. (**A**, **B**) Experiment from July 2022. Specimen injected with methylene blue. Visible lateral parts of the stained oral arm canals. The asterisk indicates an artifact due to the inclination of the needle during injection. Scale bars = 6 cm. (**C**) Experiment performed from August 2023, carried out with toluidine blue, at about 15 minutes after the injection. The lateral parts of the oral arm canals and the branches present after the dichotomy of the oral arms are recognizable. Scale bar = 8 cm. (**D**) Another experiment (August 2023), toluidine blue, after 5 min. The arrows indicate the staining of one of the inner part of an oral arm canal (artifact) and the release of dye from the subgenital cavity (perforation of the stomach floor). Scale bar = 4 cm. (**E**) July 2022, diffusion of toluidine blue at about 10 minutes through the adradial canals in an octant. the arrows indicate the perradial (black) and interradial (white) canals. The square bracket shows the widening of some canals and the corresponding canal network, which simulates a kind of “pseudo annular canal”. Scale bar = 2.5 cm. (**F**) Another experiment like (**E**), but after 30 min. The arrow indicates one interradial subgenital ostium. Scale bar = 4 cm.

It is important to underline that only a small amount of stain solution should be injected to avoid excessive pressure in the stomach, which would affect the subsequent observations. The injection should also be as central and vertical as possible. If the needle of the syringe is tilted, the dye would

be pressed into the corresponding side with strong pressure and thus mechanically "squeezed" directly into both oral arm canals (Fig 10A, 10D). Conversely, a low volume of dye and a weak pressure allow the dye to diffuse well through the gastrovascular circulation currents.

This flow is relatively fast, and it usually takes 5-7 minutes to reach the openings of the 16 distal dichotomized parts of the oral arms (Fig 10A-10C). The color remains confined to the lateral part of the oral arm canals for a period of about one hour and then slowly spreads to the proximal, inner part. Although there is no physical separation between the two flows, the centrifugal flow appears to involve the more lateral, outer part of the oral arm canals and the openings in the distal, dichotomized part of the oral arm canals. Centripetal flow appears to involve the openings in the more proximal part, the one preceding the dichotomy of the oral arm canals, and the more internal, flattened parts of the oral arm canals.

In the umbrella, the stain diffuses centrifugally through the adradial canals (Fig 10E, 10F). In this area, however, the flow rate is rather slow. A first series of experiments carried out in July 2022 with specimens collected off the coast of Piran (Slovenia) transported to the laboratory in Trieste (Italy) and acclimatized there for 2-3 days without food, had shown a very slow flow. Half an hour after injection, the adradial canals and the associated anastomoses were stained only in the proximal half and barely reached the inner “pseudo ring canal”. The pseudo annular (= ring) canal (Fig 10E) corresponds roughly to the limit of the rigid part of the umbrella, just before the bending point of the dye never reached the edge of the umbrella, regardless of the elapsed time. The same series of experiments was repeated the following year (August 2023), but this time directly in the Piran Aquarium, with specimens caught the day before. This time the dye flowed a little faster, and about half an hour after injection it reached the inner “pseudo ring canal”. However, in all the experiments we carried out (2022 and 2023), the diffusion of the dye in the anastomoses initially only affected the adradial area, then it spread slowly and scarcely in the more proximal portion of the perradial canals, but not in the interradial canals. Within half an hour, the stain has reached the inner “pseudo ring canal” (Fig 10D, 10E), and spreads towards the inter- and perradial canals. In rare cases it was

possible to see the staining of the distal network canals up to the edge of the umbrella. Normally, about 1 h or more after injection, once the staining has reached the per- and interradial canals, it begins to drain into the stomach and the adradial anastomoses become clearer again (if methylene blue was used as a dye) (Fig S3). The flow rate is rather slow, and the exchange of the volume of circulating liquid in the network of umbrella canals requires long times, especially in the distal anastomoses.

The experiment, in which one specimen was immersed in a small tank (Fig 11B) of colored seawater, showed, after leaving the jellyfish for about ten minutes, the presence of an influx involving the innermost/lower portions of the oral arm ramifications and the relative flat parts of the oral canals, and that the dye has reached the stomach. Recording the experiments was extremely difficult, mainly due to the time wasted (the stain intensity tends to reduce quickly), the stress induced by the rinsing passes, and the masking effect due to the residual dye on the jellyfish's epidermis.

Fig 11. Functional anatomy of the gastrovascular system. (**A**) Experiment from August 2023, methylene blue, as in Fig 10A, 10B. Scale bar = 4 cm. (**B**) Experiment carried out by immersing a specimen in an aquarium with stained seawater (Neutral red), see text. The arrows indicate the inner part of two of the oral arm canals. The asterisks indicate the stain in the stomach. Scale bar = 5 cm. (**C**) Onset of diffusion of toluidine staining through the adradial canals after about 7-8 minutes, subumbrellar view. Interradius (and one of the subgenital ostia, asterisk) exactly in the middle of the picture. Scale bar = 0.5 cm. (**D**) Stain extension after 10 minutes from the injection, subumbrellar view. (**E**) Exumbrellar view of a specimen injected with methylene blue, after 30 and more minutes. The main diffusion of staining is also visible in the interradial canals, but not yet in the perradial ones (arrows). Scale bar = 4 cm.

Discussion

Anatomical studies of jellyfish have historically been a traditional discipline, often constrained by significant limitations [18]. Despite general scientific advances in the 21st century, many of the techniques and descriptions used in this field date back to the 19th century and are still considered undisputedly valid.

The innovative protocol by Avian and colleagues [18], which yielded remarkable results for *Rhizostoma pulmo*, raised open questions about its potential reproducibility and applicability in other species. The success of this technique in *Cotylorhiza tuberculata* shows that it is indeed versatile and can be easily applied or adapted to other rhizostomean jellyfish, not only to young and relatively small specimens, but also to adults (diameter of 35 cm, present study).

Cotylorhiza tuberculata is a rhizostomean (Suborder Kolpophorae, family Cepheidae) jellyfish that was first described without a specific name by Macri [36] in his work on the "Polmone di mare" (now *Rhizostoma pulmo*; Macri, 1778, p. 20-22), later named and described as "*MEDUSA tuberculata"* in the XIII ed. of Systema Naturae edited by Gmelin [37]. *C. tuberculata* is relatively common in coastal Mediterranean waters and Northwest coast of Africa [38], and various studies on its biology, anatomy and life cycle are available [26,32,34,38–50]. In particular, the study by Kikinger [32] contains a description (and a fine drawing that has become a classic) of the internal anatomy of *C. tuberculata*, which we have used as a comparative term for our analyses.

Gastrovascular system anatomy

We described a more complex shape of the stomach than a simple generic rounded cavity, being the lower portion characterized by four perradial kidney-shaped extensions (Fig 2A, 2B), which occupy part of the space of the subgenital sinus (Fig 2B) and contain the more lateral parts of the four interradial folded gonads with proximal gastric cirri stripes. The inner space of the stomach and the underlying subgenital cavity are separated by a thin and delicate membrane that constitutes the floor of the stomach, on which the four gonads develop. Furthermore, we were able to show the presence

of thin extensions under the four kidney-shaped expansions of the stomach, which call into question the “traditional” [32] cruciform description of the subgenital sinus, now observed as more circular. Since the resin injection experiments performed in the subgenital sinus caused a dilation in these four areas, we suggest that the cruciform shape in living specimens is only functional and is determined by the normal physiological shape of the stomach when it contains circulating seawater (so that the four perradial areas are kept flattened by the stomach).

Another intriguing issue concerns the impression or scar that can be seen in the central area of the floor (the subumbrellar membrane) of the stomach (Fig 2B), with two opposing Ys and eight subsequent bifurcations. This scar recalls the early stages of formation of the subgenital sinus, as already observed in ephyrae - metaephyrae up to 1 cm in diameter, where four interradial invaginations develop until they fuse centrally and close the central mouth [46]. Avian [46] hypothesized that the fusion of the four subgenital cavities is symmetrical. Current observations seem to indicate rather that the first two of these pockets touch and fuse, immediately followed by the other two orthogonal ones, which explains the two central and opposite Ys. Four oral arm canals were observed, whereas the oral arms were already eight [46]. It is logical to hypothesize (the developmental stages larger than 1 cm in diameter were not observed) the extension of four other invaginations of the mesoglea into the basal pillars that would split each of the four canals into two, resulting in the dichotomies present in the scars (Fig 2B, S2 Fig).

This pattern of scars is perfectly specular to the arrangement of the central canals of the oral arms (Fig 8) that we have described. This series of canals has already been described by both Kikinger [32] and Gibbons and colleagues [38], but in a summarizing manner and with little detail. An initially quadripartite arrangement is evident, and, based on these characteristics, the planes of symmetry in this species are only two, orthogonal, and not radial. However, scar figures are not uncommon in other rhizostomeans. *Rhizostoma octopus* [51,52], for example, present a similar structure in the central subumbrellar portion of the oral arms (although, in this case, the suture is central, but below the residual proximal portion of the cruciform canal of the manubrium).

In the umbrella, we recorded a different pattern of the canal system compared to the more uniform canal topography described in Gibbons and colleagues [38], where the canals are well differentiated proximally. In our specimens, we observed numerous anastomoses between canals, branching immediately after a few millimeters from their origin at the lateral edges of the stomach. The only exceptions were the interradial canals, in which the first part (one centimeter or less) from the origin was free of ramifications (Fig 3). For the first time in *Cotylorhiza tuberculata*, we have also described an area of greater flow that appears to functionally simulate an inner ring canal (Fig 11D, 11E; S3 Fig).

In taxonomy, the number of adradial canals is a common diagnostic feature in the order Rhizostomeae. However, the resulting number in our specimens (which is difficult to count given the high degree of anastomoses present) is variable, ranging from a minimum of 7 (in younger specimens) to a maximum of 9 - 11, with different values even in different octants of the same specimen. It should also be noted that in some cases 2 canals originate from a single opening (which we counted as one). Therefore, we suggest that the number of adradial canals is not a strong diagnostic character, as the differences within the genus *Cotylorhiza* are really minimal. *C. erythraea* is described to possess 4-8 adradial canals per octant [44,51–53], while *C. ambulacrata* features 11-13 adradial canals per octant [38,51,52].

The canals of the oral arms of *Cotylorhiza tuberculata* are rather flattened in their medial part, where the gastrodermis of both sides joins, but in a rather labile manner (Fig 6A, 6B). These strips look more wrinkled than the rest of the gastrodermis, suggesting an adaptation to at least partially separate the flows between the inner and outer parts of the canals (Fig 6A, 6C), which is also evident from the flow simulation (Fig 7C). In addition, a smaller number of anastomoses (Fig 7A, 7B) can be observed in the inner part of these canals compared to the canals depicted by Kikinger [32] or Gibbons and colleagues [38].

The use of resin endocast and subsequent 3D imaging obtained from X-ray microtomographic data, allowed us to detect a series of parameters on the isolated oral arms (Table 1). In the two specimens

we analyzed (15 and 35 cm in diameter), the ED/length ratio remained constant. The similar values observed within *C. tuberculata* life stages and *Rhizostoma pulmo* (ED/length ratio of 0.82, Avian et al., 2022) may indicate a convergently evolved anatomical solution to face similar nutritional and functional demands, and ecological roles. The number of endpoints increased with size. This calculation yielded 952 and 5632 endpoints, respectively, of which approximately 900 for the small individual and 5200-5400 for the adult specimen can be assumed to be true openings. The only possible comparison with the available literature data is with *R. pulmo*, for which Avian and colleagues [18] measured 4001 openings (oral arms plus scapulae) in a specimen with a diameter of 14.6 cm. It should be considered that the examined specimen of *R. pulmo* specimen was not a fully developed adult, as this species can normally reach a diameter of 40 cm. It is therefore evident that the two taxa differ significantly in the total number of openings. This diversity must be interpreted taking into account their different feeding strategies. Both species are planktophagous, but *C. tuberculata* also feature zooxanthellae as gastrodermic endosymbionts [32,34], which provide a significant part of its energy requirements. This adaptation could also justify the reduced number of openings present on the oral arms. The exploitation of symbionts could support the much faster growth rate of *C. tuberculata* compared to *R. pulmo*. In our experiments in 2022, juvenile specimens of *C. tuberculata* (with diameters between 2-3 cm up to 10-15 cm) were collected at the end of July. Within three to four weeks, only adult specimens (with a diameter of about 30 cm) could be observed. In the case of *R. pulmo*, juvenile specimens (2-7 cm in diameter) were observed in the Grado lagoon (Italy) in July, but specimens of about 30 cm were only sighted between October and December.

Functional anatomy

In the umbrella, experiments have shown that centrifugal flows involve only the adradial canals (Fig 10D, 10E) and, once reached the pseudo-ring canal, the flow then spreads along this ring, reaching first the perradial canals (Fig 11F) and, successively, the interradial ones (S3 Fig). Then,

the dye centripetally diffused centripetally through these canals towards the stomach (S3 Fig), as commonly observed in other jellyfish [51,52,54]. It is noteworthy that circulation in the most distal part of the umbrella canal system is much slower and a complete diffusion of the dye to the umbrella margin was never observed (in a few cases, only some octants per jellyfish were totally filled). Flow speeds in the umbrella are slower in *Cotylorhiza tuberculata* (over 1 hour to partially reach the umbrellar margins), when compared to the previous research on *Rhizostoma pulmo* (about 20-30 minutes to completely reach the umbrellar margins [18]). A possible explanation could be linked to the fact that *C. tuberculata* hosts gastrodermic (=endodermic) zooxanthellae [32,34], which are distributed into the network of umbrella canals, just above the radial muscle fibers (Fig 11C). If the metabolic requirements for the pulsations are also supported by symbionts, the diffusion of liquids from the stomach up to the margins could become less important, and therefore slower. Regarding diffusion times between more and less starved individuals, no changes in flow rate were noted in the manubrium, while slower flows were observed in the umbrella of starved specimens. In both conditions, however, the dye never diffused into the entire distal portion of the umbrella canal system.

Experiments performed by injecting dyes into the stomach of living specimens (diameters between 10-15 cm) confirmed what had already been observed in *R. pulmo*, namely that the outflow occurs in the outermost part (the most dilated) of the oral arm canals (Fig 10, 11). Rarely, in the orals arm canal, the dye slowly spread from the outer to the inner canal, indicating that the adhesion area between these structures is not perfectly sealed (Fig 7C), as observed in *R. pulmo* [18], which features true hemi-canals. The immersion experiment seems to confirm the observations cited above. In this case, the colored portions of the canals are the inner ones, as is the stomach (Fig 11B), supporting the theory of a bidirectional flow (centripetal in the innermost portion, centrifugal in the outer one) induced by the beating of the gastrodermal ciliated cells in the canals of the oral arms. Therefore, also in this case, the openings on the lower surface of the oral arms are to be considered as mouthlets in the most proximal area of the oral arms before the dichotomy, while

those present in the distal portions perform the function of anuses. This circulation pattern corresponds to that of *R. pulmo* [18], but the centrifugal flow of the manubrium in *C. tuberculata* is faster.

The present observations in *C. tuberculata* seem to confirm a division of flows in the apparatus of the oral arm canals, centripetal in the medial area, and centrifugal in the distal, lateral area, as also observed in *Rhizostoma pulmo* [18]. Although these species are notably different from a morpho-anatomical point of view (in *C. tuberculata* there is no distinct longitudinal subdivision of the oral arm canals and no separation of the perradial canals in the stomach) [18], the functional pattern is quite similar.

The family Cepheidae (Infraorder Actinomyaria) currently includes five genera: *Cephea*, *Cotylorhiza*, *Marivagia*, *Netrostoma* and *Polyrhiza*. The presence of eight oral arm canals at the level of the arm disc and the regression of the central mouth (completely absent in these species, no data are available for *Marivagia*; [55–57]) can be considered as one of the diagnostic characters of this infraorder [51,52]. In the other rhizostomeans, the oral arm canals generally originate from the remnants of the central mouth. Despite these anatomical differences, the distribution of flows in the oral arm canals described in *C. tuberculata* recalls the one described in the Rhizostomatoidean jellyfish *Rhizostoma pulmo* [18].

The most recent phylogenetic reconstructions [35] indicate the Rhizostomatidae and the Stomolophidae (both Daktyliophorae) as the most phylogenetically distant taxa from the Kolpophorae (including Cepheidae). Thus, given that this circulation pattern is shared by two distantly related taxa, it is conceivable that this represents a symplesiomorphic characteristic that is more widespread within the order Rhizostomeae than previously thought. This hypothesis seems to be supported from a morphological point of view by Stiasny's [44] analysis of the oral arm canal systems of various species of Rhizostomeae, where further examples of oral arm canals with internal constriction are reported, such as *Lobonemoides robustus* (now *L. gracilis*) [44]. This additional observation of separate circulation and openings with different functions in Cnidaria

provides further evidence for a through-gut like system, which also revives the debate on the digestive system evolutionary theories [18,19].

In conclusion, we have confirmed the validity of the protocol proposed by Avian and colleagues [18], with which excellent results were also obtained in *C. tuberculata*. The ease of use, flexibility and universality of this protocol could mean that it can be applied by jellyfish researchers all over the world and is not limited to selective studies.

From an evolutionary perspective, these discoveries of the double canal and inner circulation are a strong argument in the evolutionary debate about the development of the gastrovascular system in Animalia [19,24,25,58]. We hope that our evidence will challenge traditional paradigms and help to change the perception of jellyfish as "simple" organisms on the one hand, and, on the other hand, undermine the commonly thought bulletproof evolutionary theory that proposes evolution as a straight line from ancient and simple organisms to newer and more complex organism, as unique or rare events that alter this linearity of complexity are progressively being observed over time [18,59–61].

The authors conclude with the hope that this study and Avian and colleagues [18] could serve as the basis for a new worldwide wave of descriptions and updates of the functional anatomy of jellyfish, the significance of which goes beyond Cnidarian research and the implications of which have a major impact on broader scientific debates.

Materials and methods

Sampling area

Cotylorhiza tuberculata (Macri, 1778) individuals were collected in two locations in the Gulf of Trieste (North Adriatic Sea): the LTER_EU_IT_056 site in 2022 (Italy) (Fig 1) and Piran (Slovenia) in 2022, 2023. Dates of samplings are reported in Table 2.

Fig 12. Sampling map. Square indicates the top left magnification of the Gulf of Trieste (Italy). Red triangles indicate the sampling areas.

Table 2. List of collected samples and locations for *C. tuberculata.*

Locality	Time	Nr of specimens
LTER_EU_IT_056 (Sistiana – Duino) 45.70’ N, 13.71’ E	July 20, 2022	2 adults (> 25 cm)
Piran (Slo) 45°31'54.02"S 13°33'39.33"E)	July 25, 2022	37 juveniles (5 – 20 cm)
Piran (Slo) shore 45°31'39.57"S 13°33'59.31"E	September 9, 2022	2 adults (25 cm)
Fijesa (Slo) 45°31'33.19"S 13°34'53.30"E	August 9, 2022	2 adults (> 25 cm)
Piran (Slo) shore 45°31'39.99"S 13°33'58.01"E	August 17 - 24, 2023	24 juveniles (5-20 cm)

Sampling design

Juveniles of *C. tuberculata* usually appear in the Gulf of Trieste in early summer and adults disappear when temperatures drop during late autumn. We collected specimens of all sizes and developmental stages by diving sessions using containers to avoid physical damage to jellyfish. A total of 61 juveniles (diameter up to 15 cm) and 6 adult jellyfish (diameter over 25 cm) were sampled. All research present in the manuscript (being on jellyfish) did not require ethical approval. In 2022, half of the specimens were reared in tanks (Aurelia-80, Reef-Eden), the other half were fixed in 5% formalin-saltwater solution and then stored.

Morphological analyses

Characterization of the gastrovascular system and functional anatomy

To describe *Cotylorhiza tuberculata* gastrovascular system we followed the protocol proposed by Avian and colleagues [18].

Methylene blue (0.5%), Toluidine blue (0.5%) and Neutral red (1%) were injected in-vivo from the apex of the umbrella into the stomach with an insulin syringe (S4 Fig). The internal flux circulation experiments were performed in July 14-15th, 2022, and August 17-25th, 2023, and video recorded (Canon G16 camera). When the canals were filled, the specimens were immediately fixed in 5% formalin. The oral arms were then excised and observed under a Leica Microsystems M205C Stereomicroscope equipped with a Canon G16 camera.

In a second experiment, the intake flow of *C. tuberculata* was investigated. For this purpose, for each trial, a single specimen was immersed for 10 min in a 5 L tank containing seawater dyed with 1.5% Neutral red. The specimen was then transferred in a series of three tanks filled with seawater to "clean" the outer surface and then filmed as above.

Internal cast of the gastrovascular system

We applied the protocol by Avian and colleagues [18] to make multiple casts of the whole and single parts of the gastrovascular system to obtain the clearest visualization of the ramifications of the canals. Individuals fixed with 5% formalin were used. We chose Liquidissima Resin Pro© Epoxy resin with a 5% acetone dilution to obtain the best balance between viscosity and fluidity. Fixed jellyfish were kept in water to sustain bodyweight and avoid tissues breakdown, and the resin was injected into the stomach using a 20-ml syringe for smaller specimens, and a 50-ml syringe for larger specimens. Jellyfish tissues were gently squeezed to distribute the resin throughout the gastrovascular system. The resin hardened within 24 hours, then jellyfish tissues were digested for 24 hours at 40°C in a 20% KOH solution (sample/solution volume ratio 1:5).

X-ray computed microtomography measurements

Three endocasts (two juveniles of 13 and 15 cm and one adult of 35 cm of diameter) were analyzed trough the microfocus X-ray computed tomography (µCT) technique by using the FAITH instrument of the Elettra synchrotron light facility in Basovizza (Trieste, Italy). This fully customized cone-beam CT system is equipped with a sealed microfocus X-ray source (Hamamatsu L12161, Japan) operating in a 40-150 kV energy range at a maximum current of 500 µA. For this study, a flat panel detector (Hamamatsu C11701DK) with a resolution of 2192 × 1776 pixels and a pixel size of 120 × 120 µm² was used. By adjusting the source-object-detector distance it is possible to obtain a spatial resolution close to the minimum focal spot size of the source (5 µm) for samples of different dimensions varying from 1-2 millimeters up to 30 cm in lateral size. For these acquisitions, we set the detector position in an offset configuration, which granted an approximately 60% increase in the lateral Field of View (~1.6 times the standard Field of View). Two set of scans were performed at both low (voxel size 84 µm) and medium (voxel size 36 µm) resolution.

The low-resolution X-ray µCT scans on the whole sample were acquired using the following experimental conditions (High Voltage setup):

- Source: tube voltage = 100 kVp, tube current = 270 µA, focal spot size = 20 µm, 0.2 mm Cu filter
- Geometry: source-sample distance = 420 mm, source-detector = 600 mm, equivalent voxel size 84 µm
- Offset detector geometry [62] with a displacement of 30%
- number of projections = 3600, angular step = 0.1 degrees, exposure time = 0.2 s, total scan duration = 12 min.

The medium resolution X-ray µCT scans on the whole sample were acquired using the following experimental conditions (High Voltage setup):

- Source: tube voltage = 100 kVp tube current = 270 µA, focal spot size = 20 µm, 0.2 mm Cu filter

- Geometry: source-sample distance = 180 mm, source-detector = 600 mm, equivalent voxel size 36 µm
- Offset detector geometry [62] with a displacement of 30%
- number of projections = 3600, angular step = 0.1 degrees, exposure time = 0.2 s, total scan duration = 12 min.

CERA software v. 7.0.0 (Siemens Healthineers AG, Germany) was used to reconstruct a set of 2D axial slices from the projection images (radiographs) obtained by the flat panel detector during a full 360-degree rotation of the specimen. Fiji [63] and VGStudio MAX 2.0 (Volume Graphics, Germany) were used to investigate the structure and anatomy of the three samples.

Morphometric analysis of the X-ray microtomographic data

To obtain a comprehensive description of the gastrovascular system, we analyzed specific regions of the specimen, with different foci: 1) the entire cast, 2) the entire manubrium to compare the structure of the oral arms, and 3) a single oral arm to characterize the topology of its structures (the wings) supporting the openings of the canals.

A fully quantitative analysis is essential to accurately assess differences between jellyfish samples imaged at different developmental stages. As shown by Avian and colleagues [18], the most informative parameters are derived from the skeletonization process of the oral arms. The topological characterization via skeletonization and skeleton analysis has been carried out on the most complete and accurate casted and skeletonized oral arms to avoid possible underestimations due to incomplete injection of the resin, cast fractures and, at the same time, to avoid under- or overestimation due to the skeletonization process. This approach was preferred to a complete analysis of the entire endocast as a few oral arms were not fully recovered or filled with the resin.

The analysis was performed by calculating "skeleton" for each oral arm. This skeleton in this case is defined as the medial axis of the structure (i.e. the medial axis of the gastrovascular canal structure in the oral arm) calculated using the thinning algorithm [64].

The detailed analysis of the oral arms proceeded as follows: (i) selection and isolation of the most complete oral arm from each dataset, (ii) binarization using automated thresholding [65] to prepare for skeletonization, (iii) 3D Gaussian smoothing to maintain consistent medial axis positioning, followed by segmentation again, to create a noise-reduced binary volume, avoiding the need for aggressive pruning after skeletonization, (iv) 3D hole-filling to eliminate gas bubbles in the resin, and a connected components calculation to remove "floating" objects that could interfere with skeletonization, (v) skeletonization via the thinning algorithm with two cycles of pruning for the shortest branches (3 voxels), and (vi) skeleton analysis.

All steps were executed using ImageJ/Fiji software [63], with volume renderings created using VGStudio MAX 2.2 (Volume Graphics, Germany).

The topological characterization of the systems features two main concepts: the local thickness (LT) analysis, and the "skeleton" (i.e., the medial axes of the structure, in this specific case) analysis. The LT of a segmented class in a tomographic dataset is described as the calculation for each voxel (vx) of the radius of the maximum inscribed sphere in the class that contains that vx [66]. The skeleton has been measured via the "thinning" algorithm [64], as we were interested in the calculation of the medial axis of the structures. The combination of the two analyses can provide a volume where the medial axes are labeled, vx by vx, with the LT values, to provide the local quantification of the thicknesses. A similar approach has been successfully applied to characterize the pore space topology in porous materials [67].

The analysis on both the manubrium and the single arm was carried out as follows: first, the grayscale 8-bit dataset was segmented using the Otsu thresholding algorithm [68] applied to the full dataset grayscale frequency histogram to generate a binary volume. The binarized volume was then used to calculate the LT of the segmented class (the gastrovascular system). In the dataset #1 (manubrium) two sub-datasets, the first featuring the scapulae and the second featuring the 8 (eight) oral arms, were separated and analyzed independently. In the dataset #2 (the single oral arm) the

three-winged portions were manually separated under the scapulae and subsequently independently analyzed.

The binary volumes of the different systems were filtered using an isotropic Gaussian filter with a structuring element of 5 (five) vx and followed by a segmentation preserving the original dataset connectivity to generate a smoother structure. This filtering is necessary to reduce the noise of the data and suppress the generation of many spurious branches in the skeletonization process. The 3D-isotropic-Gaussian filter fully preserves the mediality of the original dataset, while suppressing the smallest branches generated by the structure roughness during the thinning process. After the skeletonization, two consecutive 4 (four) vx pruning cycles, to delete the remaining short ending branches, were applied. This combination of filtering and pruning operations provided the smallest number of spurious end branches (e.g., the ones radiating from the long oral arm structures, generated by changes in shape and not by the presence of canals), while preserving the short branches at the oral apertures. In order to provide a thickness-labeled skeleton, each vx in the resulting skeletons was labeled using the respective LT value. The analysis of the single oral arm was carried a step further, to also investigate the number of openings: the number of endpoints (ideally equivalent to the number of oral apertures) was then calculated, separately for each wing of one oral arm.

References

1. Ahyong S, Boyko CB, Bernot J, Brandão SN, Daly M, De Grave S, et al. Cnidaria. World Register of Marine Species (WoRMS). Accessed at: https://www.marinespecies.org/aphia.php?p=taxdetails&id=1267 on 2025-03-21. WoRMS Editorial Board; 2025. Available: https://www.marinespecies.org
2. Hamilton G. The secret lives of jellyfish: long regarded as minor players in ocean ecology, jellyfish are actually important parts of the marine food web. Nature. 2016;531: 432–435.
3. Hays GC, Doyle TK, Houghton JDR. A Paradigm Shift in the Trophic Importance of Jellyfish? Trends Ecol Evol. 2018;33: 874–884. doi:10.1016/j.tree.2018.09.001
4. Delle Chiaie S. Memorie sulla storia e anatomia degli animali senza vertebre del regno di Napoli. Dalla stamperia de fratelli Fernandes; 1828.
5. Stiasny G. Das Gastrovascularsystem als Grundlage für ein neuse System in Rhizostomeen. Zool Anz. 1923;17: 241–247.
6. Stiasny G. Mitteilungen über Scyphomedusen II. Zool Meded. 1928;11: 177–198.
7. Purcell JE, Arai MN. Interactions of pelagic cnidarians and ctenophores with fish: a review. Jellyfish Blooms: Ecological and Societal Importance. Springer Netherlands; 2001. pp. 27–44. doi:10.1007/978-94-010-0722-1_4
8. Brotz L, Cheung WWL, Kleisner K, Pakhomov E, Pauly D. Increasing jellyfish populations: trends in Large Marine Ecosystems. In: Purcell J, Mianzan H, Frost JR, editors. Jellyfish Blooms IV: Interactions with humans and fisheries. Dordrecht: Springer Netherlands; 2012. pp. 3–20. doi:10.1007/978-94-007-5316-7_2
9. Condon RH, Duarte CM, Pitt KA, Robinson KL, Lucas CH, Sutherland KR, et al. Recurrent jellyfish blooms are a consequence of global oscillations. Proc Natl Acad Sci. 2012;110: 1000–1005. doi:10.1073/pnas.1210920110

10. Graham WM, Gelcich S, Robinson KL, Duarte CM, Brotz L, Purcell JE, et al. Linking human well-being and jellyfish: ecosystem services, impacts, and societal responses. Front Ecol Environ. 2014;12: 515–523. doi:10.1890/130298
11. Morandini AC. Impacts of jellyfish: gelatinous problems or opportunities? 2022.
12. Gibbons MJ, Richardson AJ. Beyond the jellyfish joyride and global oscillations: advancing jellyfish research. J Plankton Res. 2013;35: 929–938. doi:10.1093/plankt/fbt063
13. Pitt KA, Lucas CH, Condon RH, Duarte CM, Stewart-Koster B. Claims that anthropogenic stressors facilitate jellyfish blooms have been amplified beyond the available evidence: a systematic review. Front Mar Sci. 2018;5: 451.
14. Magalhães C, Martins A, Dos Santos A. New approaches to study jellyfish: From autonomous apparatus to citizen science. Zooplankton ecology. CRC Press; 2020. pp. 227–251.
15. Greene C, Kuehne L, Rice C, Fresh K, Penttila D. Forty years of change in forage fish and jellyfish abundance across greater Puget Sound, Washington (USA): anthropogenic and climate associations. Mar Ecol Prog Ser. 2015;525: 153–170.
16. Lynam CP, Lilley M, Bastian T, Doyle T, Beggs S, Hays G. Have jellyfish in the Irish Sea benefited from climate change and overfishing? Glob Change Biol. 2011;17: 767–782.
17. Stoner EW, Layman CA, Yeager LA, Hassett HM. Effects of anthropogenic disturbance on the abundance and size of epibenthic jellyfish Cassiopea spp. Mar Pollut Bull. 2011;62: 1109–1114.
18. Avian M, Mancini L, Voltolini M, Bonnet D, Dreossi D, Macaluso V, et al. A novel endocast technique providing a 3D quantitative analysis of the gastrovascular system in Rhizostoma pulmo: An unexpected through-gut in cnidaria. Plos One. 2022;17: e0272023.
19. Presnell JS. The Presence of a Functionally Tripartite Through-Gut in Ctenophora Has Implications for Metazoan Character Trait Evolution. Curr Biol. 2016;26: 2814–2820.
20. Arai MN. A Functional Biology of Scyphozoa. Springer Science & Business Media; 1997.

21. Avian M, Ramšak AC. Classes Scyphozoa, Cubozoa, And Staurozoa. In: Schierwater B, editor. Invertebrate Zoology: A Tree of Life Approach. CRC Press; 2021.
22. Ruppert EE, Barnes RD, Fox RS. Invertebrate Zoology: A Functional Evolutionary Approach. Brooks/Cole, Belmont, CA; 2004.
23. Fankboner PV. Digestive System of Invertebrates. Encyclopedia of Life Sciences. Chichester: John Wiley & Sons Ltd1–6; 2003.
24. Hejnol A, Martindale MQ. Acoel development indicates the independent evolution of the bilaterian mouth and anus. Nature. 2008;456: 382–386.
25. Hejnol A, Martín-Durán JM. Getting to the bottom of anal evolution. Zool Anz. 2015;256: 61–74.
26. Morandini A, Holst S, Reinicke G. Advances in Rhizostomeae jellyfish research. 2024.
27. Thibault D, Kuplik Z, Prieto L, Enrique-Navarro A, Brown M, Uye S, et al. Ecology of Rhizostomeae. Advances in Marine Biology. Elsevier; 2024. pp. 397–509.
28. Prieto L, Astorga D, Navarro G, Ruiz J. Environmental control of phase transition and polyp survival of a massive-outbreaker jellyfish. PloS One. 2010;5: e13793.
29. Alparslan M, Doğu S, Özalp B. Importance of Jellyfishes and Their Distribution around the Çanakkale Strait. Workshop on jellyfish and other gelatinous species in turkish marine waters 20-21 May 2011. 2011. p. 78.
30. Benovic A, Lucic D. Jellyfish outbreaks: natural cycle or stress response effect. Gelatinous zooplankton outbreaks: theory and practice CIESM Workshop Series, Monaco. 2001. pp. 59–63.
31. Gulsahin N, Tarkan AN. Seasonal distribution of Scyphozoa (Cnidaria) and Ctenophora species in Gökova Bay, Mugla, Turkey. Rapp Comm Int Mer Médit. 2013;40: 538.
32. Kikinger R. Cotylorhiza tuberculata (Cnidaria: Scyphozoa)-Life history of a stationary population. Mar Ecol. 1992;13: 333–362.

33. Pérez-Ruzafa A, Gilabert J, Gutiérrez J, Fernández A, Marcos C, Sabah S. Evidence of a planktonic food web response to changes in nutrient input dynamics in the Mar Menor coastal lagoon, Spain. Nutrients and Eutrophication in Estuaries and Coastal Waters: Proceedings of the 31st Symposium of the Estuarine and Coastal Sciences Association (ECSA), held in Bilbao, Spain, 3–7 July 2000. Springer; 2002. pp. 359–369.
34. Enrique-Navarro A, Huertas E, Flander-Putrle V, Bartual A, Navarro G, Ruiz J, et al. Living inside a jellyfish: The symbiosis case study of host-specialized dinoflagellates,“Zooxanthellae”, and the scyphozoan Cotylorhiza tuberculata. Front Mar Sci. 2022;9: 817312.
35. Gamero-Mora E, Lawley JW, Maronna MM, Stampar SN, Muhlia-Almazan A, Morandini AC. Morphological and molecular data in the study of the evolution, population genetics and taxonomy of Rhizostomeae. Adv Mar Biol. 2024;98: 361–396. doi:10.1016/bs.amb.2024.09.001
36. Macri S. Nuove osservazioni intorno la storia naturale del polmone marino degli antichi. 1778.
37. Gmelin JF. Carolia Linne Systema Naturae per regna tria naturae, secundum classes, ordines, genera, species, cum characteribus, differentiis, synonymis, locis. (ed. 13). Lipsiae Impensis Georg Emanuel Beer. 1788;1662. Available: https://www.marinespecies.org/aphia.php?p=sourcedetails&id=267994
38. Gibbons M, Morandini AC, Straehler-Pohl I, Bezio N. Identification guide to macro jellyfishes of West Africa. Food & Agriculture Org.; 2022.
39. Goette A. Untersuchungen zur Entwicklungsgeschichte der Würmer: Beschreibender Teil. 1. Voss; 1882.
40. Claus C. Untersuchungen über die Organisation und Entwicklung der Medusen. Freytag; 1883.
41. Claus C. XXII.—The Ephyrøe of Cotylorhiza and Rhizostoma, and their development into eight-armed Medusøe. J Nat Hist. 1884;13: 175–183.

42. Claus C. Elementary text-book of zoology: general part and special part: protozoa to insecta. Swan Sonnenschein; 1892.
43. Hein W. Untersuchungen über die Entwicklung von Cotylorhiza tuberculata. Z Wiss Zool. 1903;73: 302–320.
44. Stiasny G. Mittheilungen über Scyphomedusen I. Zool Meded. 1921;6: 109–114.
45. Kramp PL. Synopsis of the medusae of the world. J Mar Biol Assoc U K. 1961;40: 7–382.
46. Avian M. Biological cycle of Cotylorhiza tubercolata (Macri 1778): morphological aspects of the development from ephyra to young medusa. Workshop on jellyfish in the Mediterranean Sea. NOVA THALASSIA; 1986. pp. 47–58.
47. Kikinger R. Development cycle of Cotylorhiza tubercolata (Scyphozoa: Rhizostomae). Nova Thalass. 1986;8: 73–82.
48. Straehler-Pohl I, Jarms G. Identification key for young ephyrae: a first step for early detection of jellyfish blooms. Jellyfish Blooms: New Problems and Solutions. Springer Netherlands; 2010. pp. 3–21. doi:10.1007/978-90-481-9541-1_2
49. Leone A, Lecci RM, Durante M, Piraino S. Extract from the zooxanthellate jellyfish Cotylorhiza tuberculata modulates gap junction intercellular communication in human cell cultures. Mar Drugs. 2013;11: 1728–1762.
50. Jordano M de A, Miyake H, Nagata RM, Morandini AC. Patterns of morphological development in Scyphozoa ephyrae (Cnidaria, Medusozoa). Mar Biodivers. 2022;52: 31.
51. Jarms G, Morandini A. World Atlas of Jellyfish. 2023.
52. Jarms G, Morandini AC, Schmidt-Rhaesa A, Giere O, Straehler-Pohl I. World Atlas of Jellyfish: Scyphozoa Except Stauromedusae. Abh Verhandlungen Naturwissenschaftlichen Ver Hambg. 2019.
53. Galil BS, Gershwin L-A, Zorea M, Rahav A, Rothman SB, Fine M, et al. Cotylorhiza erythraea Stiasny, 1920 (Scyphozoa: Rhizostomeae: Cepheidae), yet another erythraean jellyfish from the Mediterranean coast of Israel. Mar Biodivers. 2017;47: 229–235.

54. Russell FS. The Medusae Of The British Isles Volume II: Pelagic Scyphozoa, With A Supplement To The First Volume Of Hydromedusae. Cambridge University Press; 1970.

55. Galil BS, Gershwin L-A, Douek J, Rinkevich B. Marivagia stellata gen. et sp. nov.(Scyphozoa: Rhizostomeae: Cepheidae), another alien jellyfish from the Mediterranean coast of Israel. Aquat Invasions. 2010;5: 331–340.

56. Galil BS, Kumar BA, Riyas AJ. Marivagia stellata Galil and Gershwin, 2010 (Scyphozoa: Rhizostomeae: Cepheidae), found off the coast of Kerala, India. BioInvasions Rec. 2013;2: 317–318.

57. Mamish S, Durgham H, Al-Masri M. First record of the new alien sea jelly species Marivagia stellata Galil and Gershwin, 2010 off the Syrian coast. Mar Biodivers Rec. 2016;9: 1–3.

58. Tamm SL. No surprise that comb jellies poop. Science. 2016;352: 1182–1182.

59. Dunn CW, Leys SP, Haddock SH. The hidden biology of sponges and ctenophores. Trends Ecol Evol. 2015;30: 282–291.

60. Nielsen C. Animal Evolution Interrelationships of the Living Phyla. Oxf Univ Press Demand. 2013;53: 1689–1699.

61. Steinmetz PRH. Independent evolution of striated muscles in cnidarians and bilaterians. Nature. 2012;487: 231–234.

62. Wang G. X-ray micro-CT with a displaced detector array. Med Phys. 2002;29: 1634–1636.

63. Schindelin J. Fiji: an open-source platform for biological-image analysis. Nat Methods. 2012;9: 676–682.

64. Lee TC, Kashyap RL, Chu CN. Building skeleton models via 3-D medial surface axis thinning algorithms. CVGIP Graph Models Image Process. 1994;56: 462–478.

65. Otsu N. A threshold selection method from gray-level histograms. Automatica. 1975;11: 23–27.

66. Dougherty R, Kunzelmann KH. Computing local thickness of 3D structures with ImageJ. Microsc Microanal. 2007;13: 1678–1679.

67. Voltolini M, M. K, H. T, Ajo-Franklin J. Visualization and prediction of supercritical CO2 distribution in sandstones during drainage: An in situ synchrotron X-ray micro-computed tomography study. Int J Greenh Gas Control. 2017;66: 230–245.
68. Otsu N. A threshold selection method from gray-level histograms. IEEE Trans Syst Man Cybern. 1979;9: 62–66.

Supporting Information

S1 Fig. *Cotylorhiza tuberculata*, interradial section of a specimen of about 15 cm in diameter. Visible the Subgenital sinus (Sgs), two of the subgenital ostia (Asterisks), the stomach (St) with part of the gonads. Arrow indicates the complex of central branchings emerging from the 2-4-8 central canal system, including the future brood-carrying filaments and some smaller club-shaped digitations.

S2 Fig. *Cotylorhiza tuberculata*, Schematic drawing of the central canal system at the base of the arm disc, immediately below the subgenital sinus.

S3 Fig. *Cotylorhiza tuberculata*, living specimen after about 1h after the injection (**Methylene blue**)**.** Exumbrellar view. Evident the stain present into the stomach, the per- and interradial canals and into the "pseudo ring canal complex" (See text). The web of adradial canals is now faded. The outermost, distal part of the anastomoses was practically unstained, apart from a small area adjacent to the perradial canal on the left.

S4 Fig. *Cotylorhiza tuberculata*, stain injection (**Methylene blue**). Two living specimens, one at the moment of the injection into the stomach, the second after some minutes from the injection.

S1 Video. Micro CT-scan 3D rendering. 360˚ animation of the manubrium endocast of the specimen of 15 cm in diameter.

S2 Video. Micro CT-scan 3D rendering. 360° animation of the endocast as above with the highlight (yellow) of the oral arm selected for the quantitative measurements, and the superimposed (red) segmented gastrovascular system.

S3 Video. Micro CT-scan 3D rendering. 360° animation of the endocast of the specimen of 35 cm in diameter.

S4 Video. Toluidine blue stain diffusion into the gastrovascular system. Specimen of 14 cm in diameter. Evident the coloration of the external portion of the oral arm canals and the branches of the dichotomized distal portions. About 10 min after injection.

S5 Video. Methylene blue stain diffusion into the gastrovascular system. Specimen of 12 cm in diameter. As above. About 5 min after coloration.

S6 Video. *Cotylorhiza tuberculata*. Adult specimen of about 30 cm in diameter, swimming in the Gulf of Trieste, clip taken on August 25, 2022. The solidity of the central portion of the umbrella is evident. The central dome is immobile, the subsequent crown (about 1/3 to 2/3 of the diameter) flexes slightly during pulsation. The only truly flexible portion is the distal part.

Figure 1

Click here to access/download;Figure;Fig 1.tif

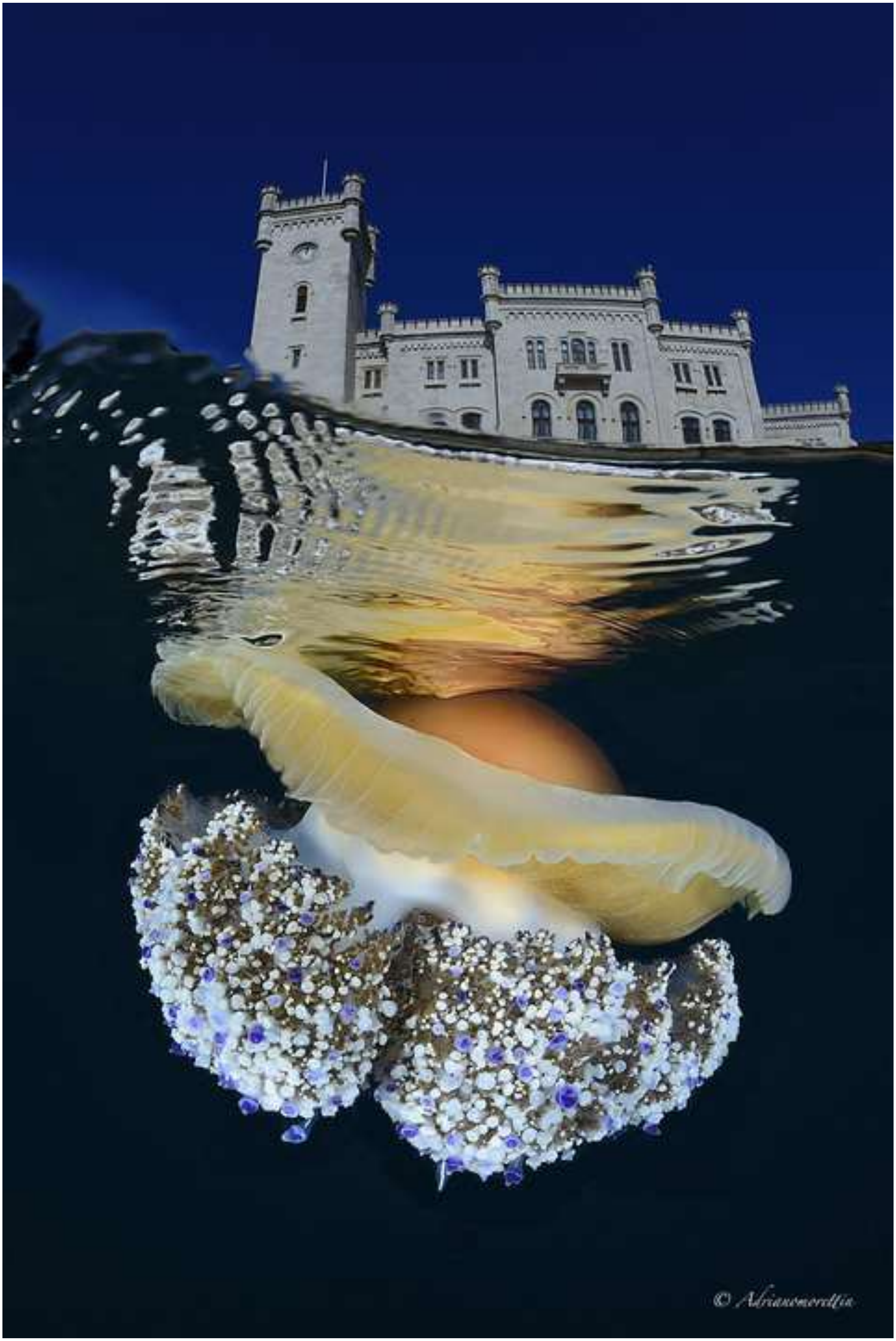

Click here to access/download;Figure;Fig 2.tiff

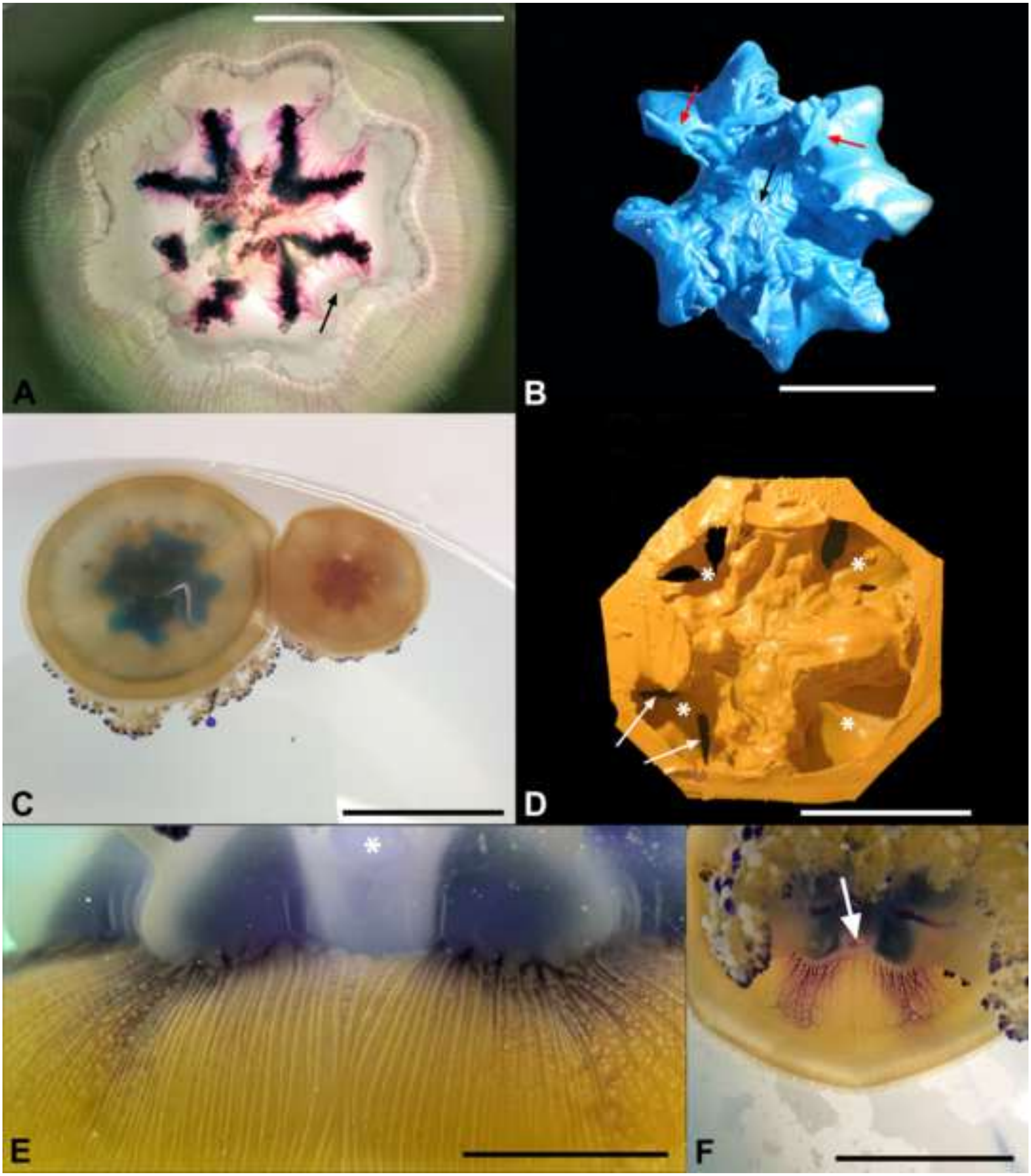

Click here to access/download;Figure;Fig 3.tiff

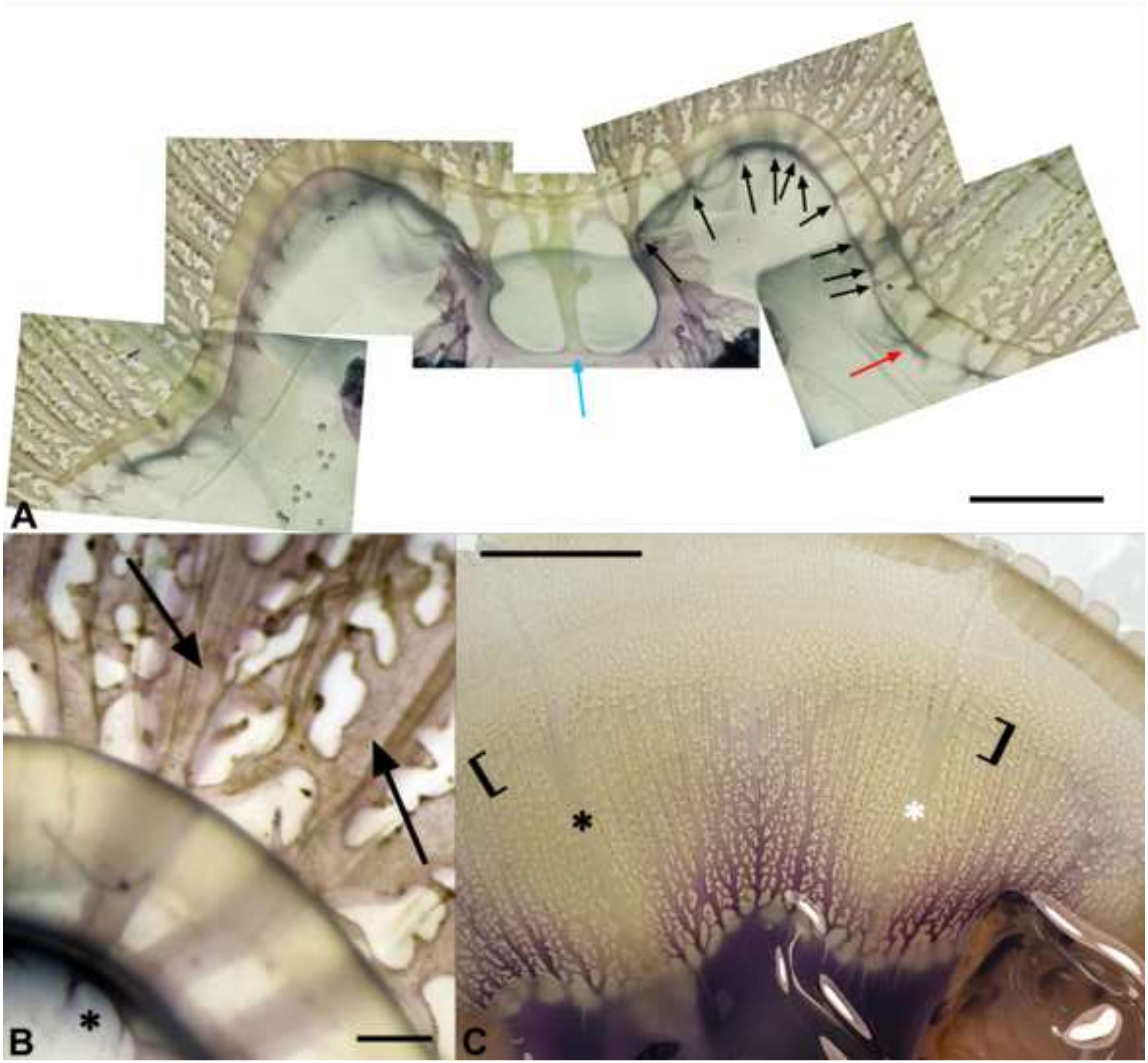

Figure 4

Click here to access/download;Figure;Fig 4.tif

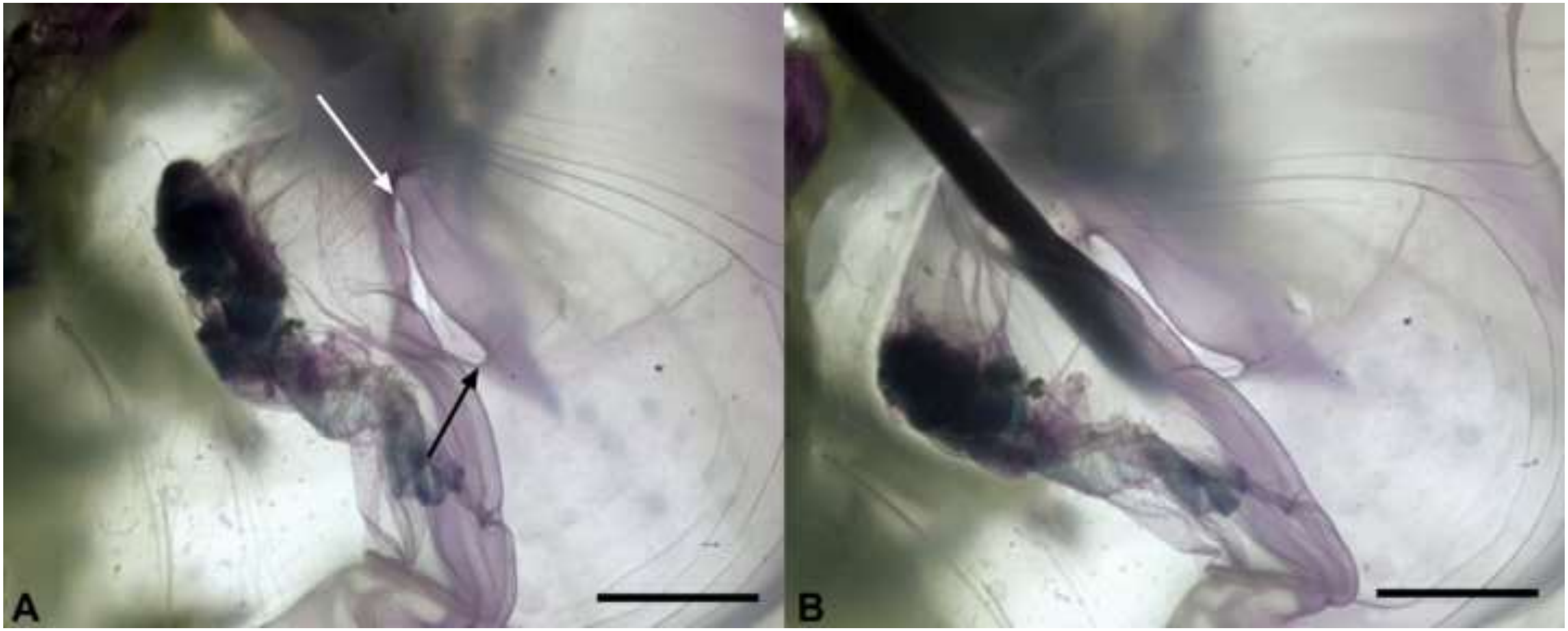

Figure 5

Click here to access/download;Figure;Fig 5.tiff

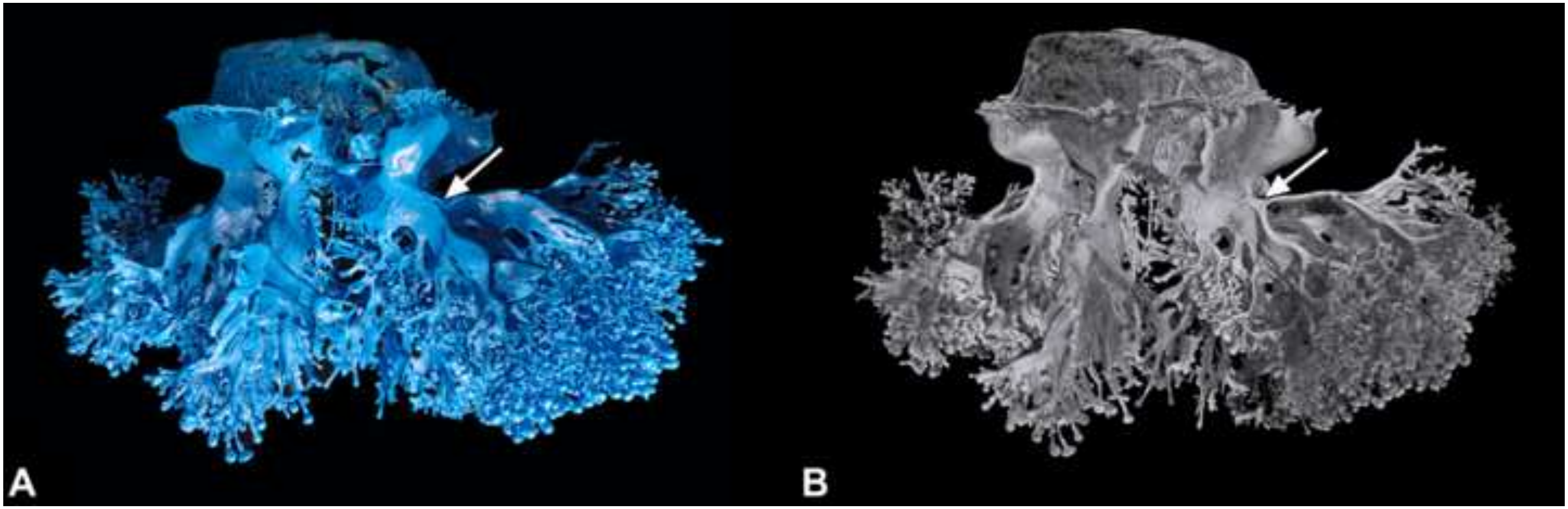

Click here to access/download;Figure;Fig 6.tif

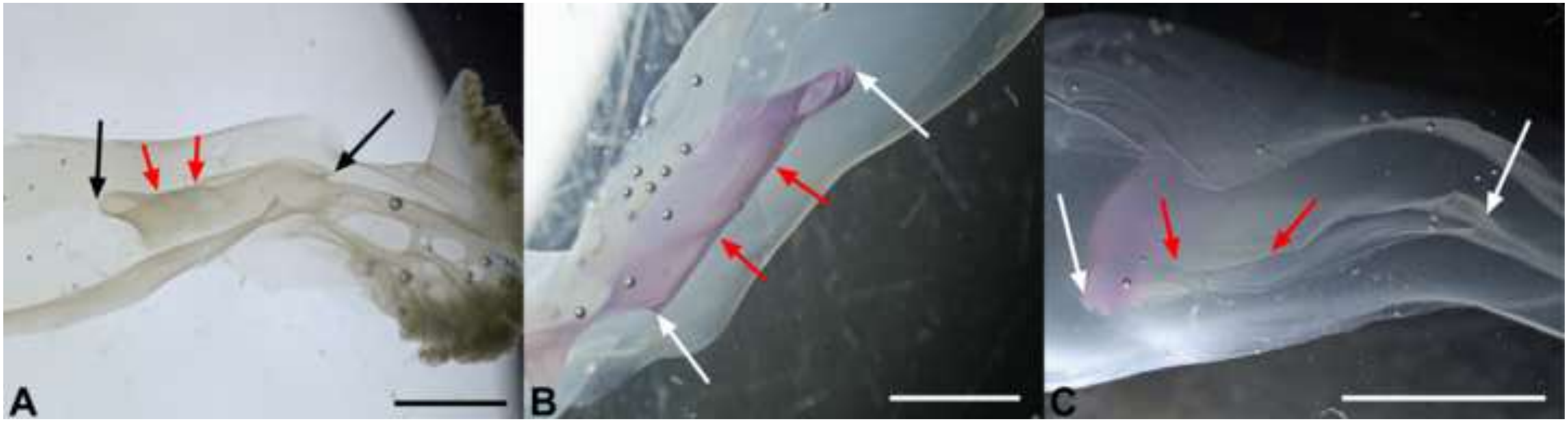

Figure 7

Click here to access/download;Figure;Fig 7.tif

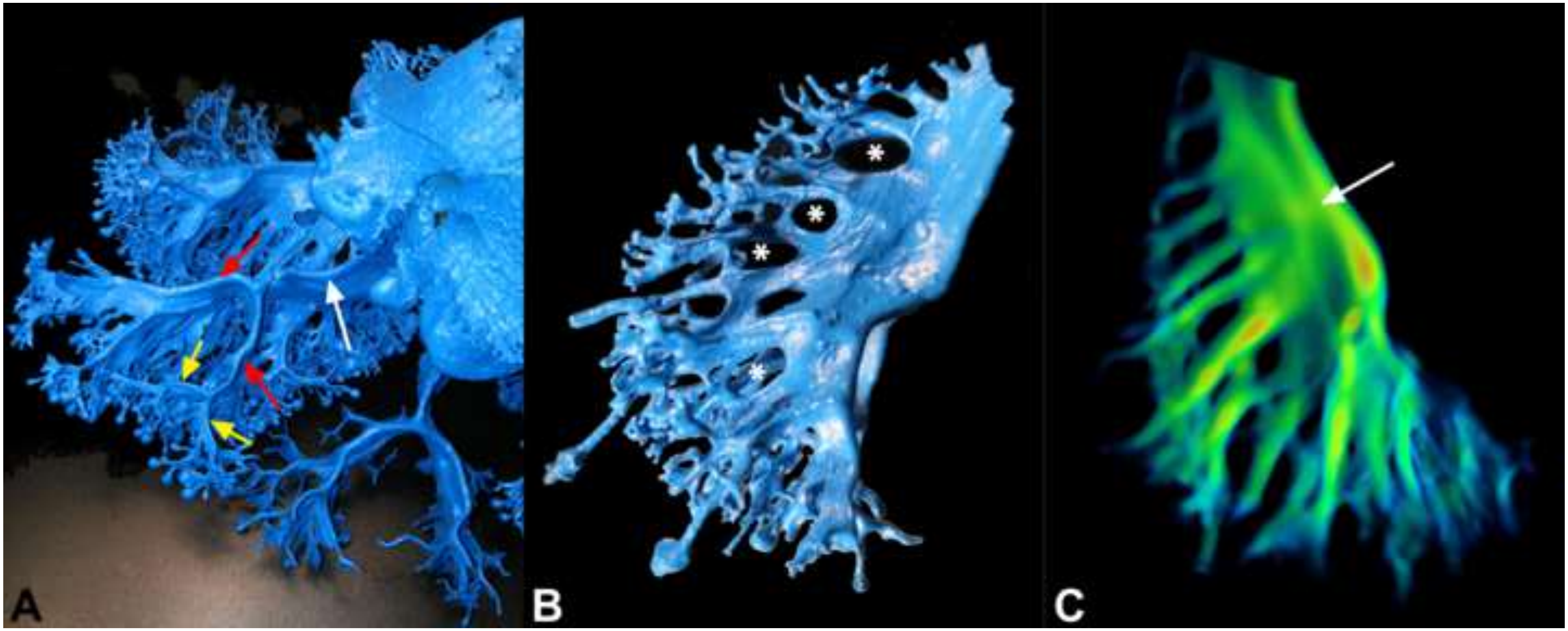

Figure 8

Click here to access/download;Figure;Fig 8.tiff

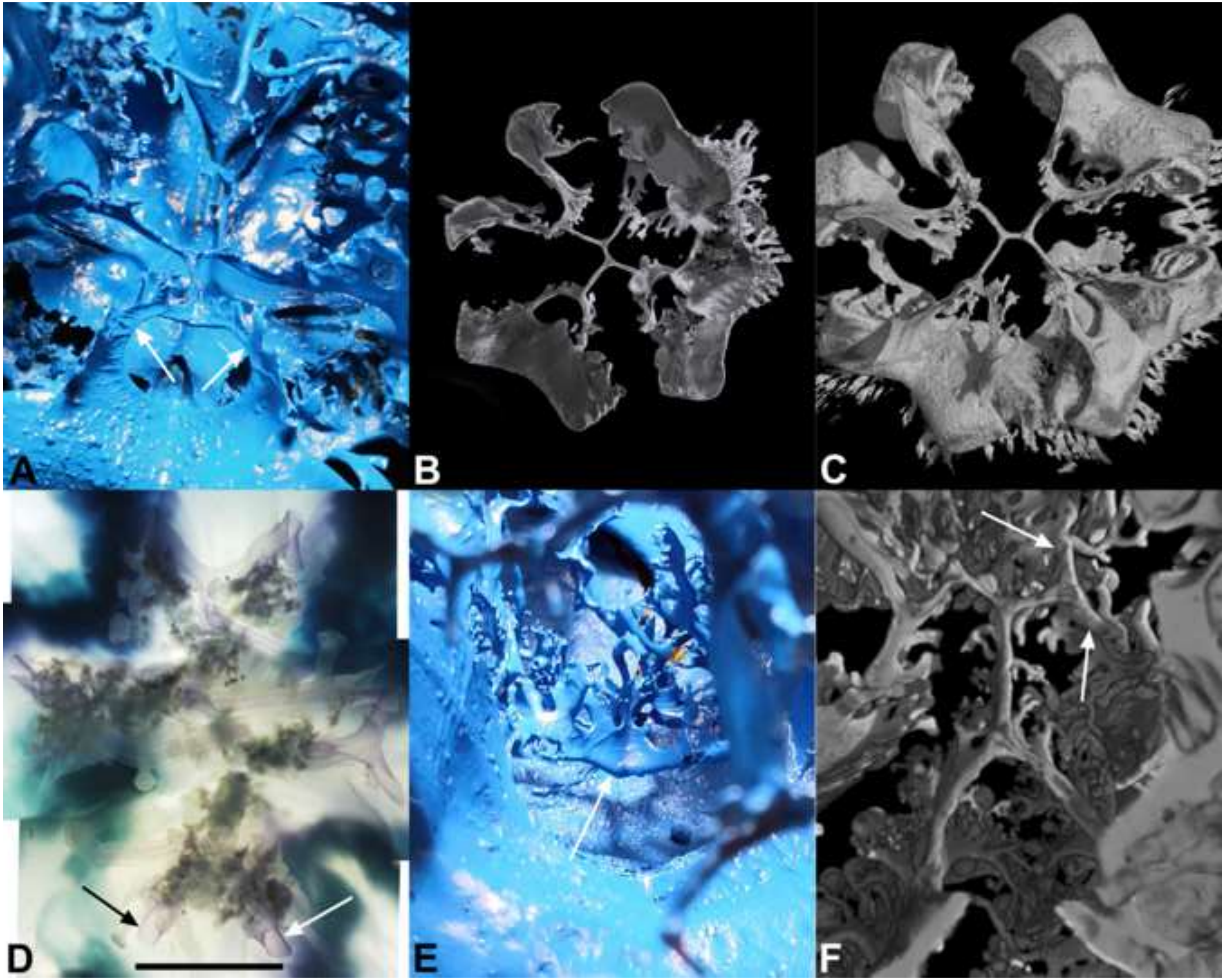

Figure 9 Click here to access/download;Figure;Fig 9.tif

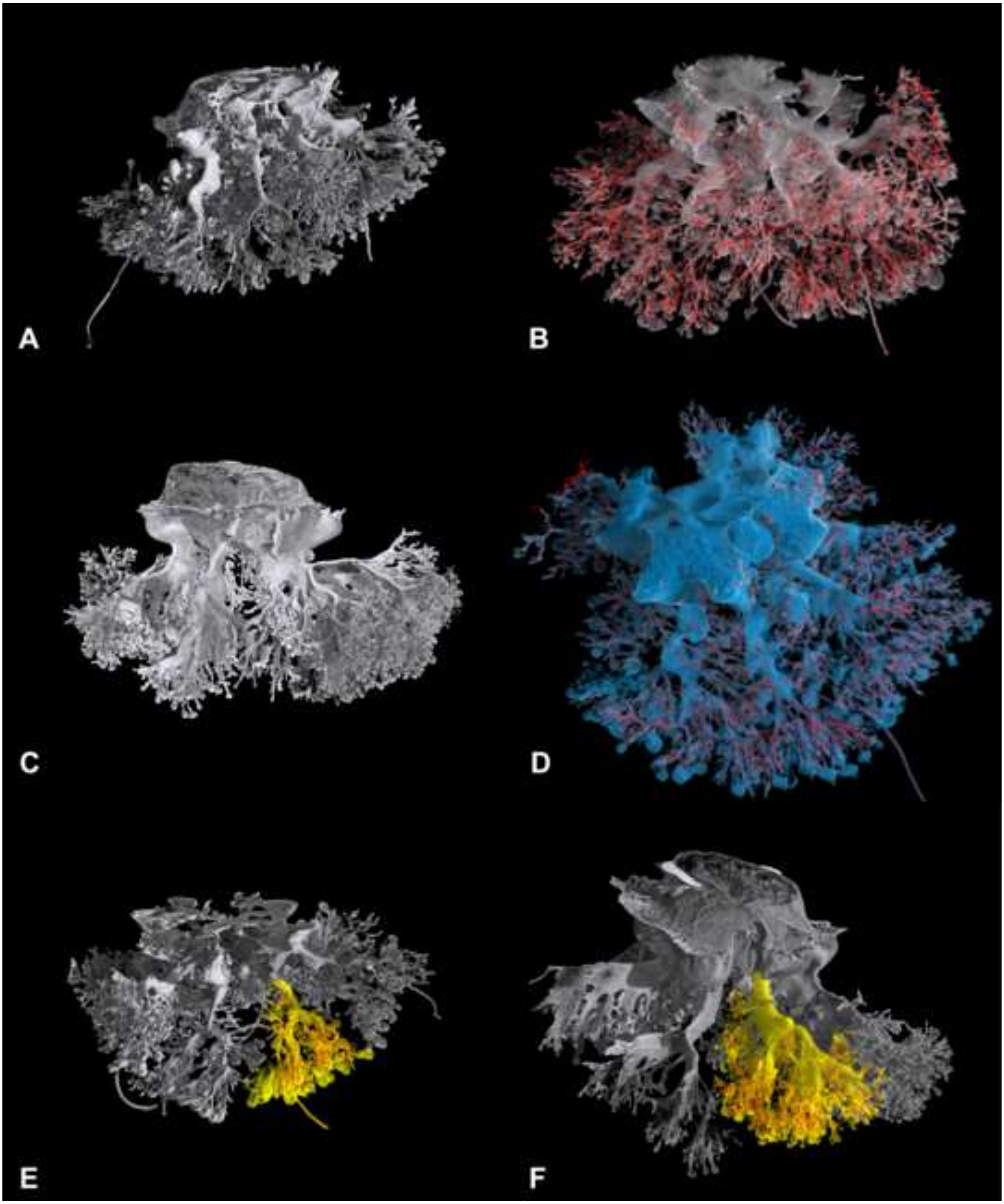

Figure 10

Click here to access/download;Figure;Fig 10.tif

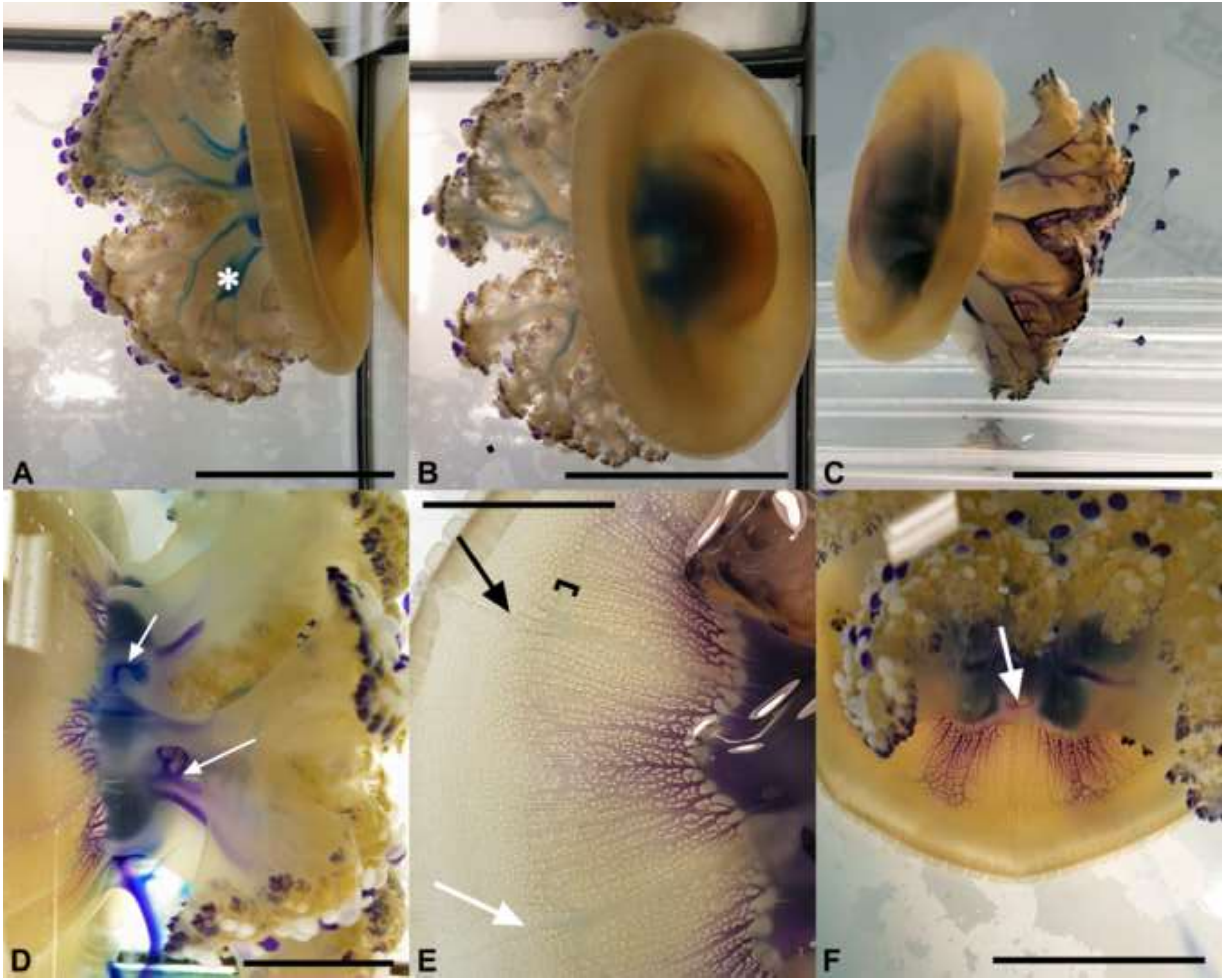

Figure 11

Click here to access/download;Figure;Fig 11.tiff

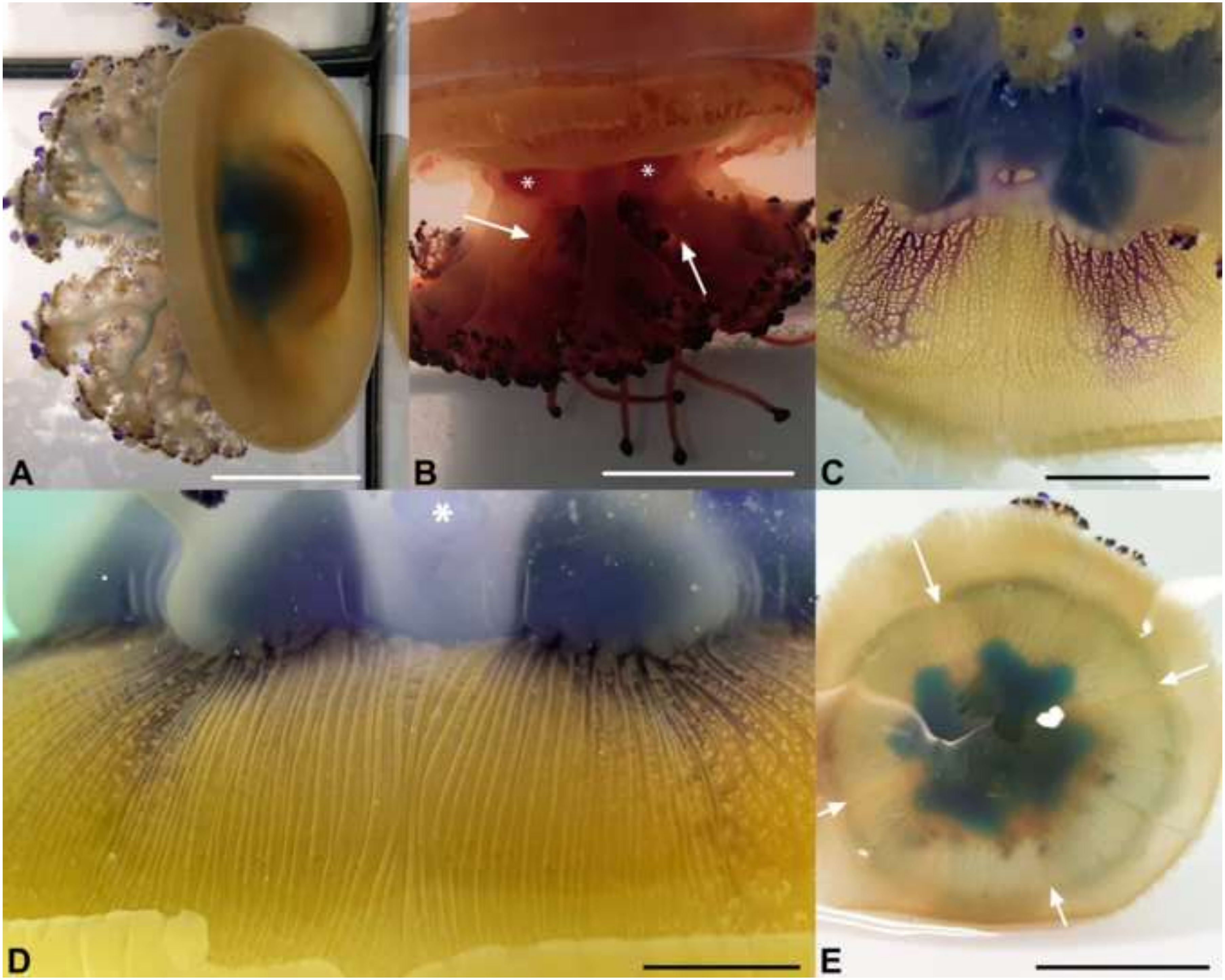

Figure 12

Click here to access/download;Figure;Fig 12.tif

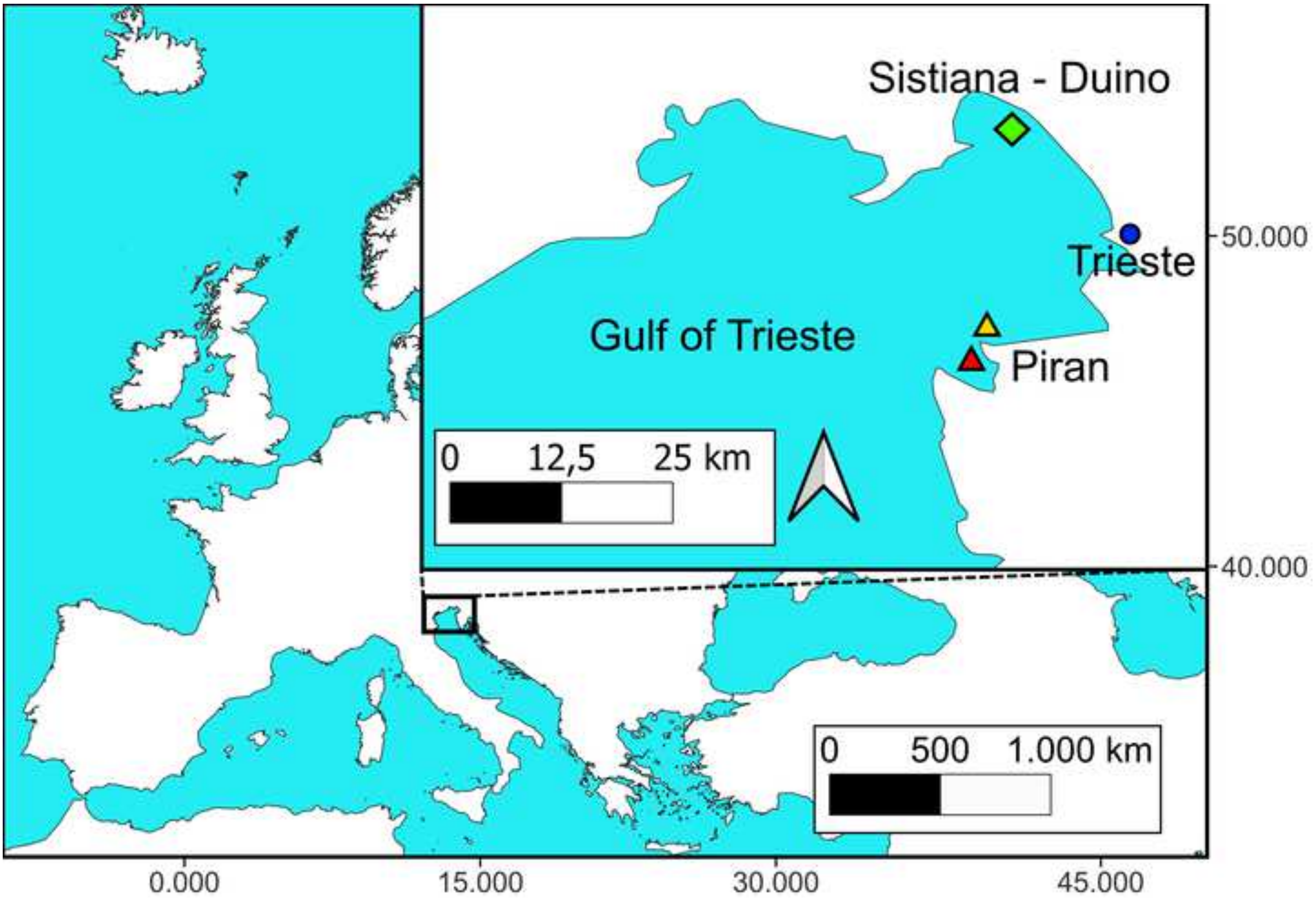

S1 Fig

Click here to access/download
Supporting Information
S1 Fig.tif

S2 Fig

Click here to access/download
Supporting Information
S2 Fig.tif

S3 Fig

Click here to access/download
Supporting Information
S3 Fig.tif

S4 Fig

Click here to access/download
Supporting Information
S4 Fig.tif

S1 Video

Click here to access/download
Supporting Information
S1 Video.avi

S2 Video

Click here to access/download
Supporting Information
S2 Video.avi

Click here to access/download

Supporting Information

S3 Video.avi

S4 Video

Click here to access/download
Supporting Information
S4 Video.mp4

S5 Video

Click here to access/download
Supporting Information
S5 Video.mp4

S6 Video

Click here to access/download

Supporting Information

S6 Video.MP4
