# Supplementary material for: New advances in jellyfish anatomy: the benefits of endocasts and X-ray microtomography in the investigation of the gastrovascular system of Cotylorhiza tuberculata (Scyphozoa; Rhizostomeae; Cepheidae)
Source: PLoS One. 2025 Nov 14;20(11):e0336682. doi: 10.1371/journal.pone.0336682 (PMC12617847; doi:10.1371/journal.pone.0336682)
Supplement: Attachment — Submitted filename: Rebuttal_Letter.docx [file pone.0336682.s011.docx]

**Editor Comments to the Author.**

Dear editor, we provide below our answers to the journal requirements.

**1.** Please ensure that your manuscript meets PLOS ONE's style requirements, including those for file naming.

**R1:** Dear editor, we checked all the style requirements, and we formatted the text as required.

**2.** In your Methods section, please provide additional information regarding the permits you obtained for the work. Please ensure you have included the full name of the authority that approved the field site access and, if no permits were required, a brief statement explaining why.

**R2:** Dear editor, no permit to access sampling sites was required because the areas are part of the public maritime domain. We added a sentence at lines 547-548.

**3.** We note that Figure 12 in your submission contain [map/satellite] images which may be copyrighted. All PLOS content is published under the Creative Commons Attribution License (CC BY 4.0), which means that the manuscript, images, and Supporting Information files will be freely available online, and any third party is permitted to access, download, copy, distribute, and use these materials in any way, even commercially, with proper attribution. For these reasons, we cannot publish previously copyrighted maps or satellite images created using proprietary data, such as Google software (Google Maps, Street View, and Earth). For more information, see our copyright guidelines: <http://journals.plos.org/plosone/s/licenses-and-copyright>.

**R3:** Dear Editor, for Figure 12, the map source is Natural Earth (Free vector and raster map data @naturalearthdata.com), which material are in the public domain, no permission to use is needed. We also uploaded the additional “other” proof file that better explains their use declaration.

**4.** Please review your reference list to ensure that it is complete and correct. If you have cited papers that have been retracted, please include the rationale for doing so in the manuscript text, or remove these references and replace them with relevant current references. Any changes to the reference list should be mentioned in the rebuttal letter that accompanies your revised manuscript. If you need to cite a retracted article, indicate the article’s retracted status in the References list and also include a citation and full reference for the retraction notice.

**R4:** Dear editor, we checked the bibliography, and it is complete and correct. We did not add or remove any citation.

**Review Comments to the Author.**

**Reviewer #1:** First, I’d like to congratulate the authors on the work. This is a very interesting study which can certainly be useful for enriching future taxonomic descriptions in Cnidaria with more anatomical details as well as revise old descriptions and contribute to studies on physiology and evolution in Animalia. The manuscript is generally well written, all the goals were achieved, and figures and supplementary material are informative and helpful. Overall, I recommend the publication of this study. However, some corrections and additional information must be added. I’m sending the PDF with my suggestions attached.

Best wishes,

Hellen

**R:** Dear Hellen, thanks for your appreciation, comments, and suggestions, which have helped us improve the quality of our work. A detailed reply to your specific comments is provided below.

**Comment 1:** Keywords should be different from the ones present in the title. Suggestion: Cnidaria, CT scan, morphology

**R1:** Dear reviewer, thanks for the comment. We agree that keywords should not match the title. We decided to keep those you suggested plus “jellyfish physiology; digestive system evolution” which are not mentioned in the title.

**Comment 2:** Line 58. Maybe replace with "bypass" or "overcome"?

**R2:** Thanks for the suggestion, we changed the term to overcome at line 58.

**Comment 3:** Line 65. Is there an extra space between these 2 words?

**R3:** We corrected and deleted the extra space at line 65.

**Comment 4:** Line 71. widely?

**R4:** Thanks for the suggestion, we changed to widely at line 71.

**Comment 5:** Line 74. Authority and year of publication should be mentioned here and deleted from Methodology section.

**R5:** We agree with the reviewer. We added the informationat lines 75-76.

**Comment 6:** Line 77. in*

**R6:** Thanks for noticing the error, we corrected at line 78.

**Comment 7:** Line 81. Authority and year of publication

**R7:** We agree with the reviewer. We added the informationat line 82.

**Comment 8:** Line 109. Indicated by the red arrows in Fig 2B?

**Comment 9:** Line 119. The figure caption informs "oral arm canal".

**Comment 10:** Line 109. Are those the "scar" mentioned in the figure caption? It doesn't sound clear to me.

**Comment 11-12:** Line 116. The caption is clear, but it contrasts with the text itself. It's confusing. Please, make the text (Lines 108-114) clearer to match the caption.

**R8-12:** Dear reviewer, thank you for your comments. We have revised the text to improve overall clarity. In particular, we added a bracket at line 110 to better define the fact that the black arrow of figure 2 indicates the Y-shaped relief structure and, accordingly, we changed the term “scar” in the figure legend (line 121) so that it now matches with the text. Plus, we added reference to the red arrows at lines 112-113 (that indicates the oral arm canals) and a white arrow with a reference in the text (line 111 and 122) to highlight the bifurcations.

We hope these changes made the text easier to read and improved the correspondence between the text and the figure legend.

**Comment 13:** Line 160-161. That's tricky because the counting may vary depending on the observer.

**R13:**

Dear reviewer, we know that counting canals may be affected by the observers’ subjectivity. For this reason, we considered counting only the openings on the margin of the stomach the most objective strategy. Attempting to count potential dichotomies (especially those that begin close to the margin) would introduce greater variability and interpretative ambiguity. Therefore, our approach should reduce observer bias and ensure consistency in the analysis.

**Comment 14:** Line 176. Delete

**R14:** We decided to keep the reference to figure 2B since it indicates the canals (red arrows) and we removed Figure 4B at line 179.

**Comment 15:** Line 180. For better visualization could you color both arrows white?

**Comment 16:** Line 181. I don't see why this is relevant. Maybe delete B.

**R15-16:** Dear reviewer, we agree with the suggestion and we deleted figure 4B. We also changed the arrow color in figure 4A.

**Comment 17:** Line 190. approximately?

**Comment 18:** Line 190. Please, write in full.

**R17-18:** We added the information (1 up to 2 cm) at line 193.

**Comment 19:** Line 190. Delete?

**R19:** Deleted the reference to figure 4A and 4B (now it is just Figure 4) at line 193.

**Comment 20:** Line 198. C as in B as in A... it's confusing. Please, be clearer.

**R20:** We agree with the reviewer about the complexity of the original caption. We have revised the whole Fig 6 caption, and we hope that clarity has been significantly improved.

**Comment 21:** Line 211. Please, color the "A" in the figure white

**R21:** Changed the color.

**Comment 22:** Line 212. Suggestion: 1st, 2nd. 3rd

**R22:** Changed as suggested at line 222.

**Comment 23:** Line 226. approximately?

**R23:** We added the info (up to 5 cm in larger specimens) at line 236.

**Comment 24:** Line 228. Delete

**R24:** Dear reviewer, we have decided to keep both Figure 5A and 5B, as they respectively show the resin cast and the micro-CT scan. This allows for a direct visual comparison, highlighting the high fidelity of the scan in reproducing even the smallest features of the original sample.

**Comment 25:** Line 245. This is not clear

**R25:** Thank you for the comment. We changed the caption of figure 8 at line 255, we hope that now it is clearer.

**Comment 26:** Line 263-264. Suggestion: [...] analysis where all canal terminations were considered endpoints.

**R26:** We agree with the comment, we changed the text as suggested at line 274-276.

**Comment 27:** Line 279. Total number of branches

**Comment 28:** Line 279. Total number of junctions

**Comment 29:** Line 279. Total number of endpoints

**R27-29:** We agree with the comments, and we changed the text as suggested in Table 1.

**Comment 30:** Line 301. Delete?

**R30:** Dear Reviewer, thank you for your suggestion. We prefer to keep Figure 10B despite its similarity to Figure 10A. The reason is that Figure 10A shows both canals in which the dye is present due to internal circulation and an example of an injection artifact (indicated by the asterisk). In contrast, Figure 10B illustrates canals where the dye has passively diffused, and it also shows the stomach's appearance after the injection.

**Comment 31:** Line 340. that we*

**R31:** Changed as suggested at line 352.

**Comment 32:** Line 342. Delete

**R32:** Changed as suggested at line 354.

**Comment 33:** Line 343. spreaded*

**R33:** Changed to past form as suggested at line 355.

**Comment 34:** Line 345. Delete

**R34:** Changed as suggested at line 357.

**Comment 35:** Line 346. began*

**R35:** Changed as suggested at line 358.

**Comment 36:** Line 346. became*

**R36:** Changed as suggested at line 358.

**Comment 37:** Line 347. was

**R37:** Changed as suggested at line 359.

**Comment 38:** Line 348. required

**R38:** Changed as suggested at line 360.

**Comment 39:** Line 348. longer*

**R39:** Changed as suggested at line 360.

**Comment 40:** Line 353. had

**R40:** Changed as suggested at line 365.

**Comment 41:** Line 362-363. Please, indicate it in the image. There is no asterisk in C.

**R41:** Dear reviewer, we noticed that we erroneously labeled figure 11C (now 11D) and 11D (now 11C). Now the caption is correct.

**Comment 42:** Line 433-434. Authority and year of publication

**Comment 43:** Line 434. Authority and year of publication

**R42-43:** Thanks for the suggestions, we added both information at lines 446-447.

**Comment 44:** Line 487. oral*

**R44:** Corrected at lines 499-500.

**Comment 45:** Line 520. Suggestion: Lobonemoides gracilis Light, 1914 (previously known as L. robustus).

**R45:** Changed as suggested at lines 532-533.

**Comment 46:** Line 536. Suggestion: along with the one by Avian and colleagues

**R46:** Changed as suggested at line 549.

**Comment 47:** Line 537. Delete

**R47:** Changed as suggested at line 550.

**Comment 48:** Line 542. Delete

**R48:** Changed as suggested at line 555.

**Comment 49:** Line 542. Please, include the total number of specimens.

**R49:** Thanks for the comment, we added the number of specimens at line 555.

**Comment 50:** Line 543. Fig 1 does not show location info. Instead, it shows the jellyfish. I suggest removing it from here and place in line 542 after "individuals".

**Comment 51:** Line 544. Fig 12 should be mentioned here.

**R50-51:** Dear reviewer, thanks for the comment. We wrote Figure 1 instead of Figure 12. We corrected the text at line 557-558.

**Comment 52:** Line 546. I found the map and caption confusing. One image is overtopping the other and there are too many shapes (circle, triangles, square) and colors (green, blue, yellow, red) that are not well explained. Please, simplify it.

**R52:** Dear reviewer, thank you for your comment. We provided a new Figure 12 caption that now explains every detail and we hope it is now less confusing.

**Comment 53:** Line 549. Length or diameter? Please, specify it in the caption.

**R53:** We added the information in the table header.

**Comment 54:** Line 549. Please, standardize the use of – (en dash) or - (dash).

**R54:** We changed the text to classic dash – in table 2.

**Comment 55:** Line 553. different sizes

**R55:** Corrected as suggested at line 575.

**Comment 56:** Line 555. Table 2 informs up to 20 cm.

**R56:** Thank you for the comment. We have now changed the size at line 577.

**Comment 57:** Line 556. (Table 2)

**R57:** Added the reference at line 578.

**Comment 58:** Line 557. What about the 24 juvelines from 2023?

**R58:** Dear reviewer, we have now added the info about 2023 individuals at lines 580-581.

**Comment 59:** Line 557. How many specimens per tank?

**R59:** Info added at line 579.

**Comment 60:** Line 563. How many mL each?

**R60:** Dear Reviewer, the injection volume depended on the size of the individual: larger animals required more dye. However, care was taken to avoid over-injection, as the goal was to fill the stomach adequately without exceeding its capacity. Overfilling could cause the dye to be pushed into underlying canals due to injection pressure rather than by the jellyfish's internal currents. We added the mL info at lines 587-589.

**Comment 61:** Line 565. How is that possible if first sampling occurred in 20 July 2022 (Table 2)?

**R61:** Thanks for the comment, we reported the wrong date. We corrected the sampling days in Table 2.

**Comment 62:** Line 583. Out of curiosity, how much does this approach cost (contrast injections + resin casts + X-ray)?

**Comment 63:** Discussing the cost-benefit of this approach seems rather relevant.

**R62-63:** Dear Reviewer, thank you for your comment. We already have reported the brand of the resin we used so it can be found on the market (it is not expensive, less than 50 Euro/L). Similarly, the stains we used are common in laboratories and do not represent a significant expense.

We did not include the cost of the scanning because there is no standardized or universally applicable pricing currently available on the market. The final cost strongly depends on several variables, including the size of the sample and the resolution required, both of which significantly influence the scanning time and, consequently, the price. In our case, we conducted the scans within the framework of a scientific collaboration, which granted us a significant discount compared to commercial/private rates. As such, the cost we incurred would not be representative or informative for other institutions, especially considering the potential variability across countries and service providers. For these reasons, we chose not to report a specific price and to discuss the costs-benefits since it would be potentially misleading and not valid worldwide.

**Comment 64:** Line 592. mm

**R64:** Corrected at line 617.

**Comment 65:** Line 593-594. Suggestion: an increase of approximately 60% in the lateral...

**R65:** Dear reviewer, this technical section was written by the physicians who performed the scan, so we prefer to retain their terminology and syntax.

**Comment 66:** Line 603. N

**R66:** Corrected at line 628.

**Comment 67:** Line 612. N

**R67:** Corrected at line 637.

**Comment 68:** Line 657. Delete

**R68:** Corrected at line 682.

**Comment 69:** Line 659. Then independently?

**R69:** Corrected at line 684.

**Comment 70:** Line 662. Delete

**R70:** Corrected at line 687.

**Comment 71:** Line 667. Delete

**R71:** Corrected at line 692.
