# Supplementary material for: New advances in jellyfish anatomy: the benefits of endocasts and X-ray microtomography in the investigation of the gastrovascular system of Cotylorhiza tuberculata (Scyphozoa; Rhizostomeae; Cepheidae)
Source: PLoS One. 2025 Nov 14;20(11):e0336682. doi: 10.1371/journal.pone.0336682 (PMC12617847; doi:10.1371/journal.pone.0336682)
Supplement: Attachment — Submitted filename: Response to Reviewers.docx [file pone.0336682.s012.docx]

Dear Editor,

We greatly acknowledge the efforts of the reviewers and the editor. All comments were carefully considered, and the manuscript was revised accordingly. As requested, we uploaded both the track-change manuscript and unmarked version. Here enclosed please find our reply to the comments from the reviewers in point-by-point fashion.

Kind regards,

Gregorio Motta

**Response to comments of Reviewer #2**

**General comment:**

Reviewer #2: The manuscript addresses interesting questions and shows how they can be answered by new modern methods and approaches. Obtained results can be of interest not only for the specialists working with scyphozoans. Most of the illustrations are of high quality and informative.

Unfortunately, the manuscript is not without certain drawback. Firstly, it is too diffuse (verbose). Secondly, some topic in discussion are not directly connected with the topic of the work and sounds fondly. Thirdly, some illustrations duplicate one another, or redundant (give no additional information).

I recommend minor revision.

**Response:** Dear reviewer, thank you very much for your appreciation and valuable suggestions. We have removed the redundant parts, and we hope the manuscript is now more concise. A detailed point-by-point reply to your specific comments is provided here below.

Following are the certain remarks for the text:

**Comment #1:** Line 22 - ..."jellyfish"... – jellyfish anatomy.

**Response:** We agree with the suggestion and we changed the text accordingly at line 22.

**Comment #2:** Line 28 - ..."adding numerous details"... - you mention only one detail...

**Response:** Dear reviewer, thanks for the comment. We decided to change the text at lines 27-30 to better define the fact that after the comma we put the focus on one of the various details.

**Comment #3:** Lines 32-33 – "These findings challenge the theory of a simple digestive system in cnidarians" - What are the doubts? That the system is "simple"? The question is in the definition of "simple system"...

**Response:** We agree with the comment. We better explained that we consider simple the traditional scheme of scyphozoan oral openings with both intake-outtake functions compared to the specialized openings we found. We added a sentence at lines 34-35 and we hope this helps to better clarify.

**Comment #4:** Line 33 – "Given the genetic distance between Cotylorhiza tuberculata and Rhizostoma pulmo,"... - With what to compare this distance? And how it affect the organization of the digestive system?
Line 35 – "...suggesting that jellyfish are more complex organisms than previously thought." - In what sense are they more complex? The plan of Cnidaria organization remains unchanged.

**Response:** Thanks for the useful comment. We understand that we have not been sufficiently clear. Here we want to stress the fact that *C. tuberculata* and *R. pulmo* belong to different suborders (thus not so near genetically speaking inside scyphozoans) but they share this similar feature, the specialized openings, which have never been described before in other jellyfish. Therefore, we suggest that maybe this anatomical characteristic may be more diffused inside the order Rhizostomeae than generally thought. We added a clarifying sentence at lines 36-39 and removed the last sentence to tone it down.

**Comment #5:** Line 56 – "...proper "internal" description of anatomy..." – proper description of "internal" anatomy.

**Response:** Corrected at line 60 as suggested.

**Comment #6:** Line 63 – " and the classic bibliography..." - What does the bibliography have to do with it?

**Response:** We agree with your comment. We changed the term bibliography, which was not the correct word, with descriptions at line 67.

**Comment #7:** Lines 68-69 – "...Cnidaria and Ctenophora have simple, single-cavity digestive systems [23], while the first through-gut appeared in Bilateria..." – it is a comparison of 'square and round'.

**Response:** Thanks for the comment. We have modified the text from line 71 to 77. We hope it is now clearer that we considered cnidarians and ctenophores together (although we are aware that they represent rather different groups), but in this context they share the feature of being positioned before bilaterians in the evolutionary tree. Importantly, in both groups, some species were demonstrated to possess more complex digestive systems (with distinct openings dedicated to ingestion or egestion) than the common single cavity.

**Comment #8:** Lines 69-71 – the last sentence of the paragraph - A fully far-fetched topic. As many cnidarians have two-directional flows in their digestive system.

**Response:** We agree with the reviewer, we removed lines 75-77.

**Comment #9:** Lines 79-80 – "...as it is widely distributed for both its abundance and distribution in the Mediterranean Sea...: - How is that?

**Response:** We noticed the error in the text and we changed it at line 85.

**Comment #10:** Line 80 – see comments to line 33.

**Response:** Dear reviewer, as for the comment at line 33 we added the reference at line 86-87 of their genetic distance to better clarify that we choose *C. tuberculata* since it is an important species in the Mediterranean Sea (and this also helps with finding enough specimen to analyze) and because this species and *R. pulmo* (which we already analyzed with cast and microCT) are not so closely related between Rhizostomeae, thus giving the opportunity to assess the potential differences and similarities inside the order Rhizostomeae.

**Comment #11:** Lines 85-86 – "Our aim was to... (2) describe the anatomy and functional anatomy of this species in three dimensions and great detail..." – there are a lot more details visible in illustrations compared to the discussed in the text.

**Response:** We understand your point. We rephrased the objective sentence for better clarity at lines 91-93 and we removed lines 93-97.

**Comment #12:** Lines 131-137 – May be it is better to move this paragraph to the material and Methods.
**Comment #13:** Lines 144-146 – the last sentence "This suggests that the subgenital..." it is discussion...
**Response:** We agree with the reviewer for both suggestions. We moved and rearranged the first one in the materials and methods at lines 688-694 and the second in the discussions at lines 452-454.

**Comment #14:** Line 164 – "flattened at the ex-subumbrellar plane" – along oral-aboral axis.

**Response:** We thank the reviewer for this correction, we changed the sentence to oral-aboral axis at line 173.

**Comment #15:** Lines 166-167 – "which have no connections" – no connection to what?

**Response:** We changed the sentence at line 176. We hope that now it is clearer that adradial and perradial canals have a lot of anastomoses along their entire length, while interradial near their origin have no anastomoses-connections with other canals.

**Comment #16:** Lines 168-169 – "...which then decreases in the last distal third..." - one canal decreases, the other one – increases after decreasing...

**Response:** Dear Reviewer, we understand that the terminal part of the canal may appear to widen; however, this portion actually corresponds to the region of the rhopalium. For this reason, we consider it more accurate to state that the canal itself ends at the narrowest point and does not expand further.

**Comment #17:** Lines 169-171 – "According to the current state of the art, the umbrellar canals pattern of C. tuberculata is described as having no annular (or ring) canals." – It is discussion + it looks like as wide canal with points of connection between floor and roof walls.

**Response:** We agree with the reviewer, we removed the sentence and we rephrased the paragraph in the discussions at lines 490-494.

**Comment #18:** Line 173 – "...larger, perpendicular canal (connected to the anastomosis network)..." – as a part of anastomosis network.

**Response:** Changed as suggested at line 182.

**Comment #19:** Lines 174-175 – "Some zooxanthellae clusters ... of the canals" – please, indicate at the Figure.
**Response:** We added a reference to figure 3A-B and a sentence about zooxanthellae at line 167 in figure 3 caption for better clarity.

**Comment #20:** Line 179 – "...slight terminal dilatations..." which dilations – it is not clear from the figure.

**Response:** We agree with the comment. We added the reference to the white arrows at line 189.

**Comment #21:** Lines 208-212, 224, 236 – Can it be an artefact? Adhesion due to fixation?

**Response:** Dear reviewer, we observed this adhesion (now changed to apposition since it is a better anatomical terminology for two tissues which are in close contact, often touching, but not fused or stuck together) in all our experiments both on fresh tissues of jellyfish and fixed samples, so we can reject the hypothesis of the artifact.

**Comment #22:** Lines 256-261 – move to Material and Methods.

**Response:** We agree and we thank the reviewer for the suggestion, we decided to remove the paragraph since it would be redundant in the materials and methods.

**Comment #23:** Lines 300-301 – "...affects only the most lateral, slightly dilated part of the oral arm canals (Fig 10A-10C; S4 and S5 Video)." – is not convincing arguments – Fig. 10A – it is Ok, but on D-C and in Videos – not clear. The canal borders are not clearly distinguishable, and in Video the stain moves first in the center of the canal. Can it depend on the amount of fluid (food)?

**Response:** Dear Reviewer, thank you for your valuable comment. As also mentioned in response to other remarks, we have revised the manuscript by adding Figure 11 illustrating the anatomical scheme of the canals in the oral arms, in order to facilitate the interpretation of the images of the internal circulation. The canal emphasized in these images corresponds to the upper/external canal, which is responsible for egestion, whereas in the lower/internal part of the oral arm—here completely transparent—the other canal is located, which is responsible for ingestion.

**Comment #24:** Lines – 318- 324 – move to the Material and methods.

**Response:** We agree with the reviewer, we moved the paragraph to lines 663-668 in the materials and methods.

**Comment #25:** Lines 327-331 – it is discussion.

**Response:** Dear reviewer, we prefer to leave this paragraph here as it provides a descriptive account of the diffusion of the stain without entering into interpretation or discussion. We believe it contributes useful context for the reader.

**Comment #26:** Lines 338-339 – "...just before the bending point of the dye never..." – some words were lost.

**Response:** Thanks for the comment, we corrected the sentence at line 388-392.

**Comment #27:** Line 346 – "...and spread towards the inter- and perradial canals." – it should be shown.

**Response:** We agree with the comment; we put better references for Figures at line 399. We also added arrows in Figure S3 and modified the figure S3 caption to better explain the circulation.

**Comment #28:** The first sentence sounds contradictory in relation to the introduction, which said that there were few works only.

**Response:** We understand the point made by the reviewer, the terms historically and traditional could misleadingly suggest that this research field was more extensively investigated and has a larger scientific community than it actually was. We changed the first sentence of the discussion at line 426, and we hope that know it is more clear.

**Comment #29:** Lines 410-412 – "It is logical to hypothesize ... the extension of four other invaginations of the mesoglea into the basal pillars ....resulting in the dichotomies present in the scars " – what is the mechanism of mesoglea extension? The result is clear, but the mechanism is farfetched.

**Response:** Dear reviewer, this paragraph refers to the work of Avian, 1986. In that case, it was possible to follow the development up to the 1-cm diameter meta-ephyra stage. One of us (Avian M.) rewrote this section to clarify the hypothetical subsequent, unfortunately undocumented development.

We changed the whole paragraph from line 462 to 469.

**Comment #30:** Lines 428-429 – reference to the Fig 11D, 11E; S3 Fig is improbable.

**Response:** We noticed the error in the reference to figures and we changed it at line 494.

**Comment #31:** Lines 430-435 - childish prattle... The authors wrote themselves, that it would be counted according to the number of openings...

**Comment #32:** Lines 436-438 – "...as the differences within the genus Cotylorhiza are really minimal. ..." - the difference is sufficient, especially the scatters of the values do not overlap.

**Response:** Dear reviewer, although the question may seem obvious, it really has some importance, given that the number and shape of the umbrellar canals are considered diagnostic characters in virtually all jellyfish identification guides and related dichotomic keys. Indeed, it is common practice to detect this characteristic simply by observing and counting canals by transparency in the mid-distal part of the umbrella (without properly checking the origins of such canals), with the risk of overestimation. We changed the paragraph from line 503 to 516 and we hope now the concept is clearer.

Regarding the potential overlap, we respectfully maintain our interpretation: we observed 7–11 canals per octant, which show partial correspondence with both *C. erythraea* (4–8 adradial canals per octant) and *C. ambulacrata* Haeckel, 1880 (11–13 adradial canals) features.

**Comment #33:** Lines 440- 442 – "These strips look more wrinkled ... and outer parts of the canals (Fig..." - The assumption is possible, but it sounds strained, requires proofing.

**Response:** Dear reviewer, we think that it can be an issue with the scarce quality of the upload before the proofs. In Figure 6 at high quality the two tissues in the apposition part are not as smooth as the same tissues when they are more distant from each other, and the wrinkled part is more evident.

**Comment #34:** Lines 442-443 – "which is also evident from the flow simulation (Fig 7C)." - The model cannot serve as proof, as it works according to the laid program. Can help in forecasting.

**Response:** We understand the comment made by the reviewer. We found the separation of flows is not completely sealed in the experiments with live specimens in which we observed that sometimes the stain diffused from one canal to the other (medial part in Fig 6B). Other specimens, used for casts, showed the same feature, highlighted by the circulation model. Thus, we know that the circulation model (made from the cast scan) alone would not serve as proof but, in our case, it confirms what was observed in dissected samples.

**Comment #35:** Lines 461-462 – "This adaptation could also justify the reduced number of openings present on the oral arms." - the correlation is not evident...

**Response:** Dear reviewer, we agree with your comment, and we rephrased the paragraph at line 536-540 to lower the tone. We do not want to provide an explanation, but it is just our assumption. Since the *Rhizostoma* specimens we analyzed were not fully mature but possessed a higher number of mouths than *Cotylorhiza*, which, having fewer, has a lower potential energetic intake yet is able to grow more rapidly, we hypothesized that a substantial proportion of its energetic needs derives from the zooxanthellae.

**Comment #36:** Lines 462-467 – The last sentences of the paragraph have no connection with the work.
**Response:** As suggested, we removed the whole paragraph.

**Comment #37:** Lines 473-474 – "as commonly observed in other jellyfish" – it is not common for all jellyfishes - completely different situation in Aurelia.

**Response:** Dear reviewer, as reported in Russell (1970, *The Medusae of the British Isles volume II: pelagic scyphozoa, with a supplement to the first volume of Hydromedusae*), who described the internal circulation in *Aurelia aurita* (scheme at page 159) which is still considered as valid nowadays, the centrifugal circulation runs along the adradial canals and the centripetal along the per and interradial canals. Here, in *C. tuberculata,* we described the same pattern. However, for better clarity and focus, we changed the sentence “as commonly observed in other jellyfish" to “as commonly observed in other scyphozoan jellyfish" and we corrected the sentence from line 553-555.

**Comment #38:** Lines 476-477 – "...was never observed (in a few cases, only some octants per jellyfish were totally filled)..." – finally: was never observed or was observed in few cases?

**Response:** We noticed the error, we decided to move this sentence which is more results than discussions to lines 388-391.

**Comment #39:** Line 494 – reference to figure 11B - is poorly visible at the figure.

**Response:** Dear Reviewer, in Figure 11B (now 12A, we switched the panels 12A and 12B to follow the order in the text) it can be observed that, unlike the other figures which clearly highlight the outer canal, in this case the latter appears empty, while most of the staining along the oral arms is concentrated in the lower/internal region. We hope that with the new anatomical scheme (Figure 11) this would be easier to understand.

**Comment #40:** Lines 502-507 – the whole paragraph is doubling of the previous discussions... The reasoning is very verbose, with repetitions.

**Response:** We agree with the reviewer, we removed lines 582-584 to avoid repetitions in the text.

**Comment #41:** Lines 513-515 – the same...

**Response:** As for comment #40, we removed lines 593-595 to avoid repetitions in the text.

**Comment #42:** Lines 524-526 - What does it have to do with the topic of the work? Far-fetched ideas... Have you ever looked at actinia or corals, how they ventilate their gastral cavity?

**Response:** Dear Reviewer, we have revised the sentence at lines 604-605. We hope it is now clearer that what we wish to emphasize is that in *C. tuberculata* each opening has a distinct function (either intake or outtake), whereas in actinians and corals the single oral opening performs both functions (we understand that lateral canals are known to interconnect the different polyps but the intake/outtake openings are the same).

**Comment #43:** Lines 531-538 – the whole paragraph is absolutely nothing. Primitive idea. No considerations about physiology. As a hydra at school learned, so everything measured from this point of view.
**Response:** We agree with the suggestion. We totally removed the old paragraph and wrote a new one which we hope now it is more focused and clearer at lines 611-623.

**Comment #44:** Lines 858 – "...some minutes from the injection..." – after the injection.

**Response:** Changed as suggested at line 975.

**Comment #45:** Line 867 – "Evident the coloration of the external portion of the oral arm canals..." – not clear, especially in the upper part.

**Response:** Dear reviewer, in Video S4 it is visible that only the upper canal in the oral arms (the one responsible for the outflow) appears filled and evident, whereas the entire lower and inner part is completely clear. We hope that the new scheme (Fig 11) we provided will better clarify the inner structure of the oral arms.

**Comment #46:** Fig. 1 – it worth replacing this beautiful collage with the jellyfish photo and scheme of organization.

**Response:** We agree with your comment. We decided to keep the first figure, but we also provided the scheme of organization in figure 11, when we discussed the internal flow in the text.

**Comment #47:** Fig. 2. "(A) Dissection" - transverse dissection.

**Response:** Changed as suggested.

**Comment #48:** Line 121 – "...white arrow indicates..." – the white arrow is visible well only after extension of the image... Maybe it is better to change its color.

**Response:** As suggested we changed the color and the size of the arrow in figure 2B. We hope that now it is evident.

**Comment #49:** Fig 3. Line 154 – "canals" – canal.

**Response:** Changed as suggested.

**Comment #50:** (B) "Detail of (A), showing a pair of adradial canals (arrows) with a single origin (asterisk)" – inconclusive (unconvincing) example, it is better visible on left side of A.

**Response:** To improve our Figure 3B we added a dotted red line highlighting the unique opening from which the two canals then depart.

**Comment #51:** Line 157 – "The square brackets show the widening of some canals..." – widening and fusion...

**Response:** Changed as suggested at line 165-166.

**Comment #52:** Fig. 5. Line 187 – "the" – The...

**Response:** Changed as suggested.

**Comment #53:** Fig. 6. Lines199-200 – "Section of another oral arm from the specimen shown in (B), with the lateral side on the left. ..." – there is no area of adhesion...

**Response:** We agree with the reviewer. Adhesion is not the correct term. We replaced it with apposition as already explained in comment 207.

**Comment #54:** Fig. 10. Line 310-311 – "The arrows indicate the staining of one of the inner part of an oral arm canal (artifact) ..." – why it is an artifact?

**Response:** Dear Reviewer, as we mentioned in the text, it may occasionally happen that when the dye is injected too forcefully into the stomach, the pressure can drive it into the canals surrounding the stomach. In this image, it can be observed that both the outer canal (responsible for the outflow) and the inner canal (responsible for the inflow) are evenly stained, thus indicating an artifact caused by the injection.

**Comment #55:** Line 313 – "the" – The...

**Response:** Changed as suggested.

**Comment #56:** Fig. 11. There is reference in the txt only to Fig. 11B, no more. Moreover, doubling of fig. 3 and 10...

**Response:** Dear reviewer, figure 11 (now figure 12) is cited in different places throughout the manuscript. Every picture is mentioned.

**Comment #57:** Fig. 12. – large map, but what new information does it contain? Can be replaced by an inset somewhere.

**Response:** Dear Reviewer, the choice of presenting a figure with both a general view of Europe and a zoom on the study area (which is clearly visible) was made to ensure that any reader from around the world who may not be familiar with European geography can more easily contextualize our study area.

**Comment #58:** S1 Fig – lines 847-848 – "Arrow indicates the complex of central branchings emerging from the 2-4-8 central canal system, ..."- not clear. It worth replacing with a scheme of organization

**Response:** Thanks for the suggestion, we added the scheme of organization as Figure 11. We also decided to keep this figure as it is. We changed the caption for better clarity.

**Comment #59:** S5 Video – beautiful, but gives no additional information except canal dichotomy.

**Comment #60:** S6 Video - the same situation – what did authors want to show?

**Response:** We agree with the reviewer, we have decided to remove Video S5, as it was redundant, and to retain Video S6, now Video S5, since it may be useful to help the reader appreciate what an adult of this species looks like. Moreover, it clearly shows the characteristic pulsation of the umbrella, with one half remaining rigid up to the flexion point (where the ring canal is located), and from there a more pronounced movement of the umbrella margin can be observed.

**Response to comments of Reviewer #3**

**General comment:**

Reviewer #3: The manuscript has already been evaluated. The authors provided adequate responses to all comments and suggestions, following most of them and presenting reasons when do not agree completely with the reviewer. This manuscript advances the knowledge of jellyfish anatomy using modern techniques and provide substantial data and information to help in the systematics of the group and physiology of the digestive system of rhizostome jellyfishes. In my opinion it is fully accepted for publication.

**Response:** We are very grateful to Reviewer #3 for the appreciation and the positive feedback. We are pleased that the reviewer found our study valuable and well-presented.
